# Detecting the small island effect and nestedness of herpetofauna of the West Indies

**DOI:** 10.1002/ece3.2289

**Published:** 2016-07-05

**Authors:** De Gao, Gad Perry

**Affiliations:** ^1^ Department of Natural Resources ManagementTexas Tech UniversityLubbockTexas79409

**Keywords:** Herpetofauna, nestedness, nonlinear regression, small island effect, threshold value, West Indies

## Abstract

To detect the small island effect (SIE) and nestedness patterns of herpetofauna of the West Indies, we derived and updated data on the presence/absence of herpetofauna in this region from recently published reviews. We applied regression‐based analyses, including linear regression and piecewise regressions with two and three segments, to detect the SIE and then used the Akaike's information criterion (AIC) as a criterion to select the best model. We used the NODF (a nestedness metric based on overlap and decreasing fill) to quantify nestedness and employed two null models to determine significance. Moreover, a random sampling effort was made to infer about the degree of nestedness at portions of the entire community. We found piecewise regression with three segments performed best, suggesting the species–area relationships possess three different patterns that resulted from two area thresholds: a first one, delimiting the SIE, and a second one, delimiting evolutionary processes. We also found that taxa with lower resource requirement, higher dispersal ability, and stronger adaptation to the environment generally displayed lower corresponding threshold values, indicating superior taxonomic groups could earlier end the SIE period and start in situ speciation as the increase of island size. Moreover, the traditional two‐segment piecewise regression method may cause poor estimations for both slope and threshold value of the SIE. Therefore, we suggest previous SIE detection works that conducted by two‐segment piecewise regression method, ignoring the possibility of three segments, need to be reanalyzed. Antinestedness occurred in the entire system, whereas high degree of nestedness could still occur in portions within the region. Nestedness may still be applicable to conservation planning at portions even if it is antinested at the regional scale. However, nestedness may not be applicable to conservation planning at the regional scale even if nestedness does exist among sampling islands from a portion.

## Introduction

Islands have been used as model systems in developing and testing theories in ecology and evolution (Brown and Lomolino 1998). The equilibrium theory of island biogeography (MacArthur and Wilson 1967), elaborating the relationship between immigration and the extinction of species to islands depending on their size and distance from the mainland (Preston 1962; MacArthur and Wilson 1963), was a recent milestone for this theme. MacArthur and Wilson's (1967) theory provided impetus for numerous studies on species–area relationships (SARs) that concern the style in which biological diversity accumulates with area and have become one of the most fundamental patterns in nature (Lomolino 2000; Dengler 2009; Triantis et al. 2012). A potentially important feature of the SAR which is termed as the small island effect (SIE), depicting an anomalous feature of species richness on islands below a certain threshold area (Triantis and Sfenthourakis 2012), was first described decades ago by Niering (1963), MacArthur and Wilson (1967) and Whitehead and Jones (1969), and popularized 50 years later by the pioneering work of Lomolino (2000), Lomolino and Weiser (2001), and Triantis et al. (2006). Although the SIE has become more and more part of the theoretical framework of biogeography and biodiversity research, there are still several shortcomings in studies of the SIE, given the relatively short time since the patterns' recognition and the limited number of studies addressing it.

First, existing SIE studies are usually flawed in some way (Dengler 2010); thus, it is seriously criticized and its existence is even challenged in recent years (Dengler 2010; Tjørve and Tjørve 2011; Wang et al. 2012). Typically, the SIE is detected by comparing uncorrected *R* ^2^ value of regression models with and without SIE rather than by accounting for model complexity (Lomolino and Weiser 2001; Gentile and Argano 2005). This method may be seriously flawed as it is inadmissible to apply uncorrected *R* ^2^ value as a criterion for selecting the best model from candidates with different number of fitted parameters (Loehle 1990; Quinn and Keough 2002; Dengler 2010). Other potential flaws in methodology include exclusion of islands without species and not including a wide range of different candidate models (Dengler 2010).

Second, piecewise regression with two‐segment approach has been widely applied to find the upper limit of the SIE in the literature (Lomolino and Weiser 2001; Gentile and Argano 2005; Dengler 2010; Wang et al. 2012; Matthews et al. 2014; Morrison 2014). However, Lomolino and Weiser (2001) and Rosenzweig (2004) distinguished three periods at structuring the SAR: (1) SIE on small islands; (2) extinction/immigration and other ecological factors associated dynamics on islands of intermediate size; and (3) in situ speciation on large islands. Therefore, the two‐segment approach may have limitations on delimiting three SARs.

Finally, the concept of SIE is not appropriately discussed in light of recent literatures. Dengler (2010) established the terminology SIE sensu stricto, describing situations that species richness varies independently of island size below a certain threshold area, and the terminology SIE sensu lato, describing situations that the SAR slope for small islands is flatter but not necessarily zero. But, Dengler's remarks have been posteriorly criticized by Triantis and Sfenthourakis (2012) who noted that the precise meaning of the term SIE remains unresolved, as does the explanation for the phenomenon and even whether it exists; and the use of terms such as “SIE sensu stricto” and “SIE sensu lato” could further complicate the overall discussion.

Due to the unresolved shortcomings and ununified concept in studies of the SIE, taxon‐ and system‐dependent threshold values have received very limited attention. Despite the dispute, here, we applied regression‐based analyses, including linear regression and piecewise regressions with two and three segments, to detect the SIE, mainly focusing on where the slope changes among taxa, and tried to provide new insights to contribute to the still insufficiently known SIE.

Another important concept in determining inclusive distribution pattern on (true or habitat) islands is nestedness, depicting a scene in which species occurring at species‐poor islands are always present in a more species‐rich island (Patterson and Atmar 1986). Since Darlington (1957) described nested patterns, numerous studies have investigated nestedness in a wide range of taxa on both islands and fragmented habitats (e.g., Patterson and Atmar 1986; Perry et al. 1998; Fischer and Lindenmayer 2005; Schouten et al. 2007), using a variety of metrics to quantify the level of nestedness. Debate is ongoing among these metrics, each with different bias (Atmar and Patterson 1993; Wright et al. 1998; Almeida‐Neto et al. 2008; Ulrich et al. 2009). The nestedness metric based on overlap and decreasing fill (NODF) proposed by Almeida‐Neto et al. (2008) is currently considered one of the most appropriate nestedness metrics (Almeida‐Neto et al. 2008; Ulrich and Almeida‐Neto 2012; Wang et al. 2013; Matthews et al. 2015). The NODF metric allows nestedness to be calculated independently of matrix size or shape (Almeida‐Neto et al. 2008; Morrison 2013). Meanwhile, the other metrics applied in much of the previous work on nestedness have been criticized as inappropriate, and after recalculation, nestedness is thought to be less common than previously reported (Matthews et al. 2015).

However, the fact of nestedness or antinestedness is important for strategic conservation planning because it contributes to the “single large or several small” debate (Ovaskainen 2002) and the minimum set problem (Watson et al. 2011), informing protected area placement and design in fragmented landscapes (Triantis and Bhagwat 2011). Moreover, speciation occurring within large islands could lead to species endemism, decreasing the likelihood of nestedness in a system (Whittaker and Fernández‐Palacios 2007). However, if large islands are excluded and species richness is mainly governed by extinction/immigration dynamics, nestedness pattern could possibly occur according to the classical island biogeography theory (MacArthur and Wilson 1967; Patterson and Atmar 1986; Kadmon 1995). To date, although there are numerous nestedness studies, patterns in a whole system are predominantly studied, while patterns in portions of a system are almost overlooked.

**Figure 1:**
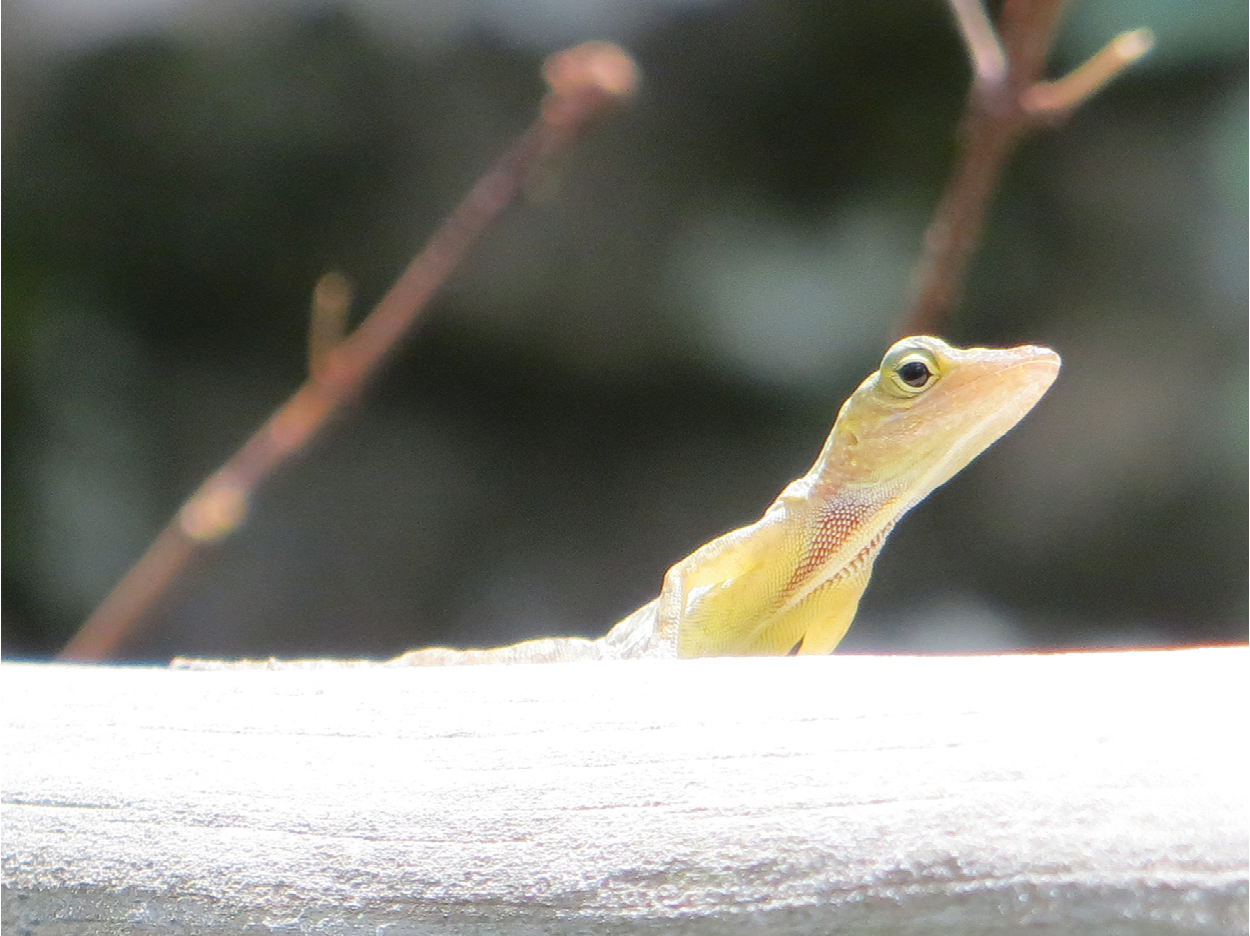
Saddled anole (*Anolis stratulus*) on a fallen tree trunk, Guana Island of the British Virgin Islands. Photograph by De Gao, October 2013.

The West Indies is a biodiversity hot spot (Myers et al. 2000), especially for amphibians and reptiles (Fig. 1). Over 90% of the herpetofaunal species in the region are endemic, sometimes even to isolated areas within an island (Hedges 2001). In order to understand the biogeographic patterns of herpetofauna in this entire region, we aim to investigate: (1) whether the SARs possess two area thresholds instead of one; (2) how the threshold values vary among taxonomic groups; and (3) whether the community composition of herpetofauna is nested in the whole or portion of the West Indies.

## Methods

### Study area and data

**Figure 2:**
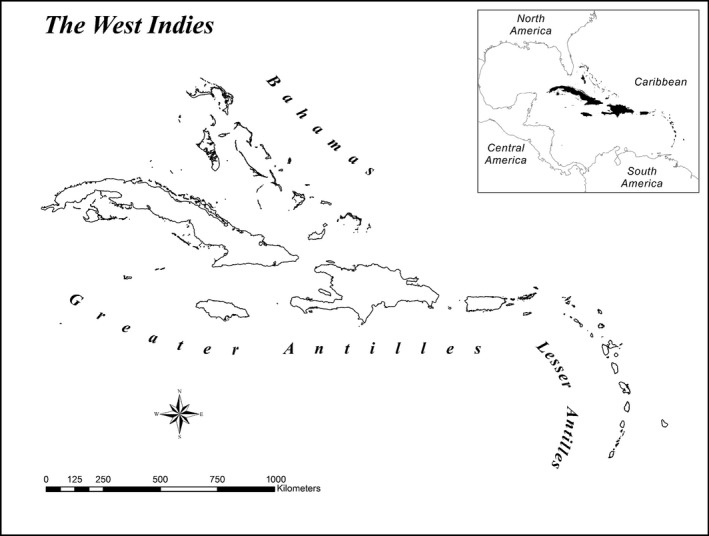
Map of the West Indies, showing the distribution of 1668 studied islands.

The West Indies comprises over 3000 islands, cays, and emergent rocks belonging to three main island groups: Bahamas, Greater Antilles, and Lesser Antilles. We derived complete herpetological species lists for each island from Powell and Henderson (2012), who recorded over 1000 species on 749 islands. We digitized islands using base maps in ArcMap 10 and ArcGlobe 10 (ESRI, Redlands, CA), including not only the 749 islands included in Powell and Henderson (2012) but also hundreds of small explored islands that have no herpetofaunal species, for a total of 1668 islands varying in area by over 10 orders of magnitude, from 3.9 × 10^‐5^ km^2^ to 1.1 × 10^5^ km^2^ (Fig. 2). The resulting map was projected by a UTM_18N coordinate system with WGS_1984 datum.

The species records were classified into three superior taxonomic groups: reptiles, *Anolis* lizards, and *Eleutherodactylus* frogs; and three corresponding inferior taxonomic groups: amphibians, *Sphaerodactylus* lizards, and *Peltophryne* frogs, respectively. As compared with the inferior groups, taxa in the superior groups are better adapted to the environment and therefore more likely to survive. Most amphibians have skins that provide little barrier to evaporative water loss, so they appear to balance their water budgets on a timescale from hours to days. In contrast, reptiles have less permeable skins and their timescale to balance the water budgets can range from days to months (Pough et al. 1998). Compared with *Sphaerodactylus* lizards that can only be found at ground level, *Anolis* lizards, as a famous case of adaptive radiation, are more ecologically adaptive, with species adapted to use different parts of the structural environment, such as ground, grass, twigs, tree trunks, and the canopy, in correspond with their morphological differences (Losos 2009). Compared with *Peltophryne* frogs, frogs in the genus *Eleutherodactylus*, which comprise the dominant frog fauna of the West Indies, contain terrestrial‐breeding frogs that lay eggs on land or tree leaves, and these eggs later hatch into miniatures of the adults, bypassing the tadpole stage, and that reproductive mode can occur in a cave, on a mountain top, or high in a tree without direct dependency on water; this greatly enhances viability in a water‐deficient and space‐limited environment (Hedges 1993).

### Regression‐based detection of the SIE

To detect the SIE, a variety of break point regression models have been applied (Dengler 2010). Among these models, the left‐horizontal with one threshold function (eq. (1)) proposed by Lomolino and Weiser (2001), defining a situation where species richness varies independently of area below a certain threshold and the two‐slope function (eq. (2)) proposed by Gentile and Argano (2005), defining a situation where the SAR slope for small islands is flatter but not necessarily zero are most widely used. These two models, however, possess only one threshold. Here, we introduce another two models with two thresholds: the left‐horizontal with two thresholds function (eq. (3)) and the three‐slope function (eq. (4)). To examine the existence and upper limit of the SIE, we compared the four break point regression models (eqs. (1), (2), (3), (4)) with the power model (eq. (5)). We used the power function as the basic function mainly for three reasons. First, it is widely used in most SIE studies (Gentile and Argano 2005; Triantis et al. 2012; Wang et al. 2012). Second, it usually fits the island SAR well (Dengler 2009; Triantis et al. 2012). Third, its model parameters have biological significance (Martín and Goldenfeld 2006; Triantis et al. 2012). In our data set, there are few very large islands outbidding the small ones in either species richness or area, which potentially generates the outlier effect, so regression analyses were fitted in log *S*‐space to ensure continuity and normality (Davies and Gather 1993; Barnett and Lewis 1994).

(1) $$\log S=c_{1}+\left(\log A>T_{1}\right)z_{1}\left(\log A-T_{1}\right),$$

(2) $$\begin{array}{cc} \log S=\; & \left(\log A\leq T_{1}\right)\left(c_{1}+z_{1}\log A\right)\\ & +\left(\log A>T_{1}\right)\left(c_{2}+z_{2}\log A\right), \end{array}$$

(3) $$\begin{array}{cc} \log S=\; & c_{1}+\left(\log A>T_{1}\;\text{AND}\;\log A\leq T_{2}\right)z_{1}\left(\log A-T_{1}\right)\\ & +\left(\log A>T_{2}\right)\left(c_{2}+z_{2}\log A\right), \end{array}$$

(4) $$\begin{array}{cc} \log S=\; & \left(\log A\leq T_{1}\right)\left(c_{1}+z_{1}\log A\right)\\ & +\left(\log A>T_{1}\;\text{AND}\;\log A\leq T_{2}\right)\left(c_{2}+z_{2}\log A\right)\\ & +\left(\log A>T_{2}\right)\left(c_{3}+z_{3}\log A\right), \end{array}$$

(5) $$\log S=c_{1}+z_{1}\log A.$$

In these equations, *S* stands for species richness, *A* for area, while *c*_*i*_ (intercept), *z*_*i*_ (slope), and *T*_*i*_ (break point) are fitted parameters. The logical AND operator combines two logical operands that have a value true or false. The expression combined by logical AND evaluates to true if both operands log *A *> *T*_1_ and log *A *≤ *T*_2_ evaluate to true; if either or both of the operands for the logical AND operator are false, the result of the expression is false. The logical expressions in brackets return value 1 if they are true and 0 if they are false. In this analysis, all 1668 islands including a large number of small ones that have no species record were involved in each taxonomic group, and species richness for islands that have no species record was log‐transformed as log (*S *+* *1) since log 0 is undefined.

**Figure 3:**
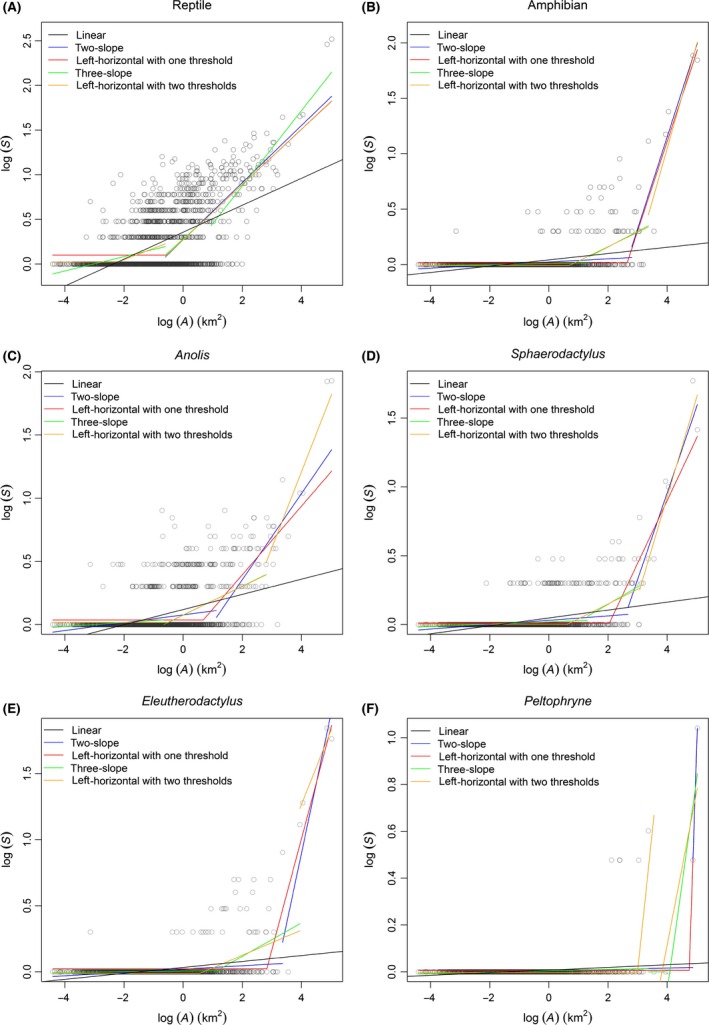
Sample results of break point and traditional species–area regression analysis for six taxonomic groups: (A) reptiles, (B) amphibians, (C) *Anolis* lizards, (D) *Sphaerodactylus* lizards, (E) *Eleutherodactylus* frogs, (F) *Peltophryne* frogs. Five different models were fitted to each taxonomic group in log *S*‐space.

We used a minimum residual sum of squares (RSS) method to estimate the threshold values (minimum RSS value will provide a maximum *r* ^2^, as *r* ^2^ = 1 − (RSS/SS total)). For equations (1), (2), (3), (4), the parameters were estimated using nonlinear estimation procedures based on iteration. Because equation (1) is continuous and break point lying between two adjacent data points will influence the RSS value of the model, we incremented the break point values (*T*_1_) by 0.001 and ran 9439 regressions for each taxonomic group (Fig. S1). Equation (2) is discontinuous and break point lying between two adjacent data points will not influence the RSS value of the model, so we assigned the break point values (*T*_1_) to the log‐transformed area values of each island and ran 1667 regressions for each taxonomic group (Fig. S2). Equation (3) is continuous at *T*_1_ but discontinuous at *T*_2_, so we assigned the second break point values (*T*_2_) to the log‐transformed area values of each island, and at any particular value of *T*_2_, *T*_1_ was incremented from the minimum log‐transformed area value to *T*_2_ by 0.001. We recorded the minimum RSS value produced by the iteration of *T*_1_ for each particular value of *T*_2_, so that the second break point (*T*_2_) was determined prior to the first one (*T*_1_). After *T*_2_ was determined, we run iteration of *T*_1_ again to look for the *T*_1_ that produced the minimum RSS value (Fig. S3). Equation (4) is discontinuous, so we assigned the first break point values (*T*_1_) to the log‐transformed area values of each island, and at any particular value of *T*_1_, *T*_2_ was assigned to the log‐transformed area values between *T*_1_ and the maximum log‐transformed area value. We recorded the minimum RSS value produced by the iteration of *T*_2_ for each particular value of *T*_1_, so that the first break point (*T*_1_) was determined prior to the second one (*T*_2_). After *T*_1_ was determined, we run iteration of *T*_2_ again to look for the *T*_2_ that produced the minimum RSS value (Fig. S4). Sample results for all regressions are graphed in Figures 3 and S5.

The Akaike's information criterion (AIC) was applied as a criterion for model selection (Burnham and Anderson 2002). For each model in each taxonomic group, we calculated the log‐likelihood (log *L*), which was used to determine AIC. For the model selection, we calculated the difference in AIC (∆AIC) and Akaike weights (*ω*) for all models to evaluate each model's probability of providing the best explanation of the data.

### Detection of nestedness

Islands that have no species record were excluded, and the presence–absence matrices were created for *Anolis* lizards (571 islands), *Sphaerodactylus* lizards (373 islands), *Eleutherodactylus* frogs (119 islands), and *Peltophryne* frogs (12 islands) (Table S1–S4). To quantify nestedness in our data sets, we used the NODF metric as it is widely regarded as the most robust (Almeida‐Neto et al. 2008; Morrison 2013; Strona and Fattorini 2014; Matthews et al. 2015). NODF scores range from 0 (no nestedness) to 100 (perfect nestedness) and increase as nestedness increases.

To determine whether an observed NODF value is significantly different from values expected for a randomly assembled community, there are many null models to choose from. The least constrained binary null model is the equiprobable–equiprobable (EE) model, which does not constrain the marginal totals and lets individuals float within the matrix, but keeps the occurrence constrained (Ulrich 2006), and has been criticized as inappropriate due to an inflation of type I error (Ulrich et al. 2009). In contrast, the fixed–fixed (FF) model that constrains matrix size, fill, marginal totals, and frequency is the most constrained binary null model (Ulrich et al. 2009). Because the FF model is more conservative and more of the original elements are retained, it is evaluated as appropriate null algorithms (Ulrich et al. 2009; Matthews et al. 2015). However, although the FF model decreases the occurrence of type I error, it may therefore increase the risk of type II error (Ulrich and Gotelli 2007). To ensure that our results were not biased owing to null model choice, we examined the significance of nestedness using not only the FF model but also the cored–cored (CC) model suggested by Beckett et al. (2014). The CC model conserves features of shape and fill, an intermediate between the EE and FF models in constraint.

Although a number of studies have tested nestedness for many taxa in a system, to our knowledge no study has tested nestedness within both whole and portion of a system. If species assemblage in a system is antinested, we are also interested in whether nestedness may still occur in any portion within the system. Thus, we conducted the following steps to calculate NODF scores of possible portion within the system for each group:

1. Randomly choose n columns (islands) from the presence–absence matrix (Table S1–S4) and form a new matrix, in which, 3 ≤ *n* < the complete island number (571, 373, 119, and 12 for *Anolis* lizards, *Sphaerodactylus* lizards, *Eleutherodactylus* frogs, and *Peltophryne* frogs, respectively).
2. Check if there are “0” rows in the new matrix. If there are, then delete them.
3. Check if the newly formed matrix is filled wholly by “1”. If the matrix is composed by “1” only, then assign “0” to the NODF score for this sampling. Otherwise,
4. Order the presence–absence matrix by decreasing number of islands occupied by each species (rows) from top to bottom and decreasing number of species present (columns) from left to right.
5. Calculate NODF score.
6. Repeat step 1–5 for 10^10^, 10^8^, 10^6^, and 10^4^ times for *Anolis* lizards, *Sphaerodactylus* lizards, *Eleutherodactylus* frogs, and *Peltophryne* frogs, respectively.

We performed all analyses using R 3.1.1 (R Development Core Team 2014). We used the vegan package (Oksanen et al. 2013) for NODF calculation and applied FALCON package (Beckett et al. 2014) to test nestedness significance. Traditionally, the number of null matrices used to make up the ensemble is fixed by the user. This method is effective providing that the ensemble is large enough to have statistical power. In the literature, authors usually use 1000 null models in their ensembles (e.g., Wang et al. 2010; Morrison 2013; Matthews et al. 2015) without concerns about undersampling or oversampling. In contrast, FALCON includes a bootstrap method for adaptive determination of ensemble size to ensure robust statistics and minimal computational load (Beckett et al. 2014).

## Results

### Regression‐based analyses for detection of the SIE

After accounting for model complexities, model selection based on AIC identified the left‐horizontal with two thresholds function (eq. (3)) as the most parsimonious model (∆AIC = 0) for *Peltophryne* frogs and the three‐slope function (eq. (4)) as the most parsimonious model (∆AIC = 0) for the rest groups (Table 1). In contrast, there was no support for the left‐horizontal with one threshold function (eq. (1)), the two‐slope function (eq. (2)), and the power function (eq. (5); all ∆AIC ≥ 11.91; Table 1). According to Akaike weights (*ω*), the chance that the left‐horizontal with two thresholds function was the best among the tested models was overwhelming (*ω *= 100%) for *Peltophryne* frogs. And the chance that the three‐slope function was the best among the tested models was overwhelming (all *ω *≥ 96%) for reptiles, amphibians, *Anolis* lizards, *Sphaerodactylus* lizards, and *Eleutherodactylus* frogs (Table 1).

**Table 1:** Results of break point and traditional species–area regression analyses of herpetofauna on 1668 islands in the West Indies

Group	Model^a^	Parameter estimate								Model selection				
		*c*_1_	*c*_2_	*c*_3_	*z*_1_	*z*_2_	*z*_3_	*T*_1_	*T*_2_	*K*	log (*L*)	AIC	∆AIC	*ω*
Reptiles	Linear	0.357			0.151					3	−257.13	520.26	238.91	0.00
	Two‐slope	0.244	0.271		0.080	0.310		−0.603		6	−140.63	293.26	11.91	0.00
	Left‐horizontal 1	0.099			0.309			−0.564		4	−174.02	356.03	74.68	0.00
	Three‐slope	0.244	0.280	0.031	0.080	0.297	0.421	−0.603	0.946	9	−131.67	281.35	0.00	0.96
	Left‐horizontal 2	0.021	0.271		0.127	0.310		−2.215	−0.603	7	−136.96	287.92	6.57	0.04
Amphibians	Linear	0.043			0.028					3	1404.93	−2803.86	1336.67	0.00
	Two‐slope	0.024	−2.163		0.014	0.828		2.804		6	1994.28	−3976.57	163.96	0.00
	Left‐horizontal 1	0.015			0.808			2.639		4	1939.10	−3870.20	270.33	0.00
	Three‐slope	0.009	−0.118	−2.720	0.004	0.139	0.941	1.354	3.365	9	2079.27	−4140.53	0.00	1.00
	Left‐horizontal 2	−0.009	−2.720		0.129	0.941		0.675	3.365	7	2063.55	−4113.10	27.43	0.00
*Anolis* lizards	Linear	0.119			0.060					3	731.14	−1456.28	451.82	0.00
	Two‐slope	0.077	−0.326		0.031	0.340		1.123		6	927.93	−1843.86	64.24	0.00
	Left‐horizontal 1	0.036			0.271			0.681		4	887.71	−1767.42	140.68	0.00
	Three‐slope	0.048	0.075	−1.193	0.016	0.114	0.600	−0.652	2.804	9	963.05	−1908.10	0.00	0.96
	Left‐horizontal 2	0.018	−1.193		0.113	0.600		−0.515	2.804	7	957.81	−1901.62	6.48	0.04
*Sphaerodactylus* lizards	Linear	0.048			0.029					3	1571.18	−3136.35	1025.87	0.00
	Two‐slope	0.031	−1.557		0.016	0.628		2.676		6	2035.03	−4058.06	104.16	0.00
	Left‐horizontal 1	0.010			0.456			2.049		4	1987.58	−3967.15	195.07	0.00
	Three‐slope	0.020	−0.104	−1.970	0.009	0.126	0.724	1.302	3.069	9	2090.11	−4162.22	0.00	1.00
	Left‐horizontal 2	0.003	−1.970		0.107	0.724		0.625	3.069	7	2072.27	−4130.55	31.67	0.00
*Eleutherodactylus* frogs	Linear	0.033			0.022					3	1586.63	−3167.26	1388.83	0.00
	Two‐slope	0.021	−3.330		0.013	1.056		3.365		6	2218.10	−4424.20	131.89	0.00
	Left‐horizontal 1	0.023			0.840			2.735		4	2168.49	−4328.98	227.11	0.00
	Three‐slope	0.005	−0.136	−0.980	0.003	0.126	0.561	1.302	3.947	9	2287.04	−4556.09	0.00	1.00
	Left‐horizontal 2	−0.012	−0.980		0.095	0.561		0.555	3.947	7	2250.32	−4486.63	69.46	0.00
*Peltophryne* frogs	Linear	0.008			0.006					3	3066.04	−6126.08	1208.52	0.00
	Two‐slope	0.004	−17.596		0.003	3.711		4.871		6	3639.96	−7267.92	66.68	0.00
	Left‐horizontal 1	0.006			3.710			4.742		4	3523.12	−7038.24	296.36	0.00
	Three‐slope	0.004	0.000	−3.702	0.003	0.000	0.905	3.365	3.947	9	3580.26	−7142.52	192.08	0.00
	Left‐horizontal 2	−0.004	−2.506		1.203	0.655		2.995	3.555	7	3674.30	−7334.60	0.00	1.00

Model performance is assessed using Akaike information criterion (AIC)‐based model selection among a set of candidate models. For each model, the fitted parameters (*c*,* z,* and *T*), the log‐likelihood (log *L*), number of estimable parameters (*K*), Akaike's information criterion (AIC), Akaike differences (∆AIC), and Akaike weights (*ω*) are presented. *T* is log10 of the area in km^2^ of the break point.

a Left‐horizontal 1 refers to left‐horizontal with one threshold; left‐horizontal 2 refers to left‐horizontal with two thresholds.

Piecewise regressions with three segments were always better than those with two segments, suggesting the existence of two break points (*T*_1_ and *T*_2_) in the data sets. We compared both *T*_1_ and *T*_2_ values between superior and inferior taxonomic groups and found *T*_1_ values were smaller in superior taxonomic groups than in inferior taxonomic groups either by the three‐slope method (reptiles −0.603 vs. amphibians 1.354; *Anolis* lizards −0.652 vs. *Sphaerodactylus* lizards 1.302; *Eleutherodactylus* frogs 1.302 vs. *Peltophryne* frogs 3.365) or by the left‐horizontal with two thresholds method (reptiles −2.215 vs. amphibians 0.675; *Anolis* lizards −0.515 vs. *Sphaerodactylus* lizards 0.625; *Eleutherodactylus* frogs 0.555 vs. *Peltophryne* frogs 2.995) (Table 1). Except for the comparison between *Eleutherodactylus* frogs and *Peltophryne* frogs, we also found *T*_2_ values were smaller in superior taxonomic groups than in inferior taxonomic groups either by the three‐slope method (reptiles 0.946 vs. amphibians 3.365; *Anolis* lizards −2.804 vs. *Sphaerodactylus* lizards 3.069) or by the left‐horizontal with two thresholds method (reptiles −0.603 vs. amphibians 3.365; *Anolis* lizards 2.804 vs. *Sphaerodactylus* lizards 3.069) (Table 1).

We compared the slope of the first segment (*z*_1_) between the three‐slope method and the two‐slope method and found the *z*_1_ parameters were all significantly different from zero (all *p *<* *0.01) either in the two‐slope approach or in the three‐slope approach for each taxonomic group. We also found that *z*_1_ values in the three‐slope approach were generally lower than those in the two‐slope approach. For example, *z*_1_ had a decrease of 71.4, 48.4, 43.8, and 76.9% from the two‐slope approach to the three‐slope approach for amphibians, *Anolis* lizards, *Sphaerodactylus* lizards, and *Eleutherodactylus* frogs, respectively.

### Results of nestedness survey

Considering all four taxonomic groups in the entire region, NODF values tended toward the antinested end of the NODF spectrum; that is, the values were much closer to 0 than 100 (mean value = 13.463; range = 3.562–35.088) (Table 2). Besides, NODF values for the four groups were not significantly lower than the means of randomly generated matrices under either the FF null model or the CC null model, which indicates that the species compositions of all four taxa have antinested structure (Table 2).

**Table 2:** Summary of results obtained from calculation of NODF (a nestedness metric based on overlap and decreasing fill) for *Anolis* lizards, *Sphaerodactylus* lizards, *Eleutherodactylus* frogs, and *Peltophryne* frogs in the West Indies

Group	Number of Species	Number of islands	Fill (%)	NODF	NODF_max_	*P*	
						FF	CC
*Anolis* lizards	285	571	0.71	7.636	100.000	0.898	0.094
*Sphaerodactylus* lizards	163	373	0.95	3.562	75.000	0.919	0.918
*Eleutherodactylus* frogs	170	119	1.65	7.567	100.000	0.935	0.999
*Peltophryne* frogs	15	12	19.44	35.088	88.889	0.927	0.392

Given are observed NODF values, the maximum NODF values obtained from random sampling (NODF_max_), and Monte Carlo‐derived probabilities that the matrix was randomly generated under null model FF and CC.

**Figure 4:**
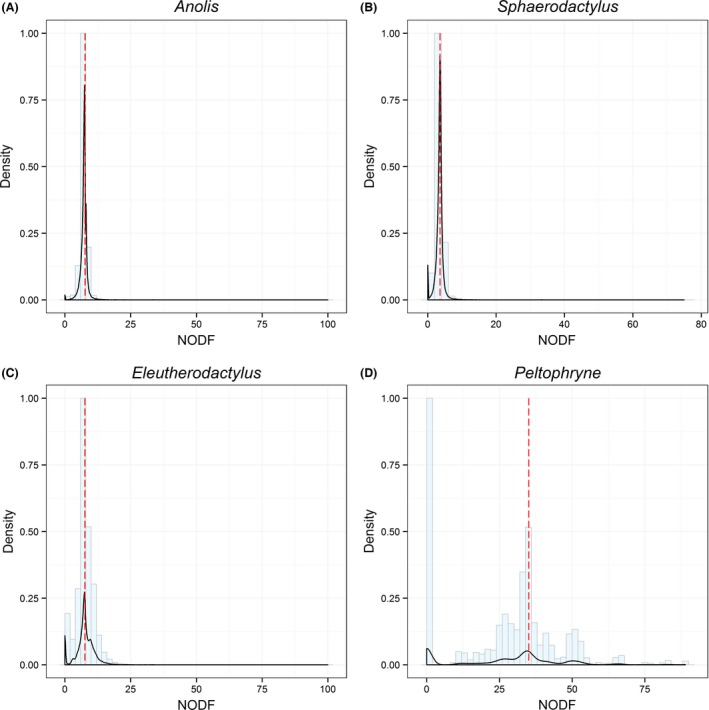
The histogram of NODF (a nestedness metric based on overlap and decreasing fill), an index for nestedness, derived from random sampling for (A) *Anolis* lizards, (B) *Sphaerodactylus* lizards, (C) *Eleutherodactylus* frogs, and (D) *Peltophryne* frogs. In each group, density is scaled to maximum of one. The red dashed line indicates the NODF score calculated for the entire system of each group.

Considering any possible portion of islands within the region, NODF values tended toward both ends of the NODF spectrum (range = 0–100, 0–75, 0–100, and 0–88.889 for *Anolis* lizards, *Sphaerodactylus* lizards, *Eleutherodactylus* frogs, and *Peltophryne* frogs, respectively) (Fig. 4), indicating that even if the species compositions in the whole system have antinested structure, nested pattern is likely to occur in some portions of the system.

## Discussion

To date, although there are a number of SIE and nestedness studies, surveyed objectives are predominantly focused at a high taxonomic level, such as plants, birds, and mammals, with fewer than 200 (habitat or true) islands in each case. Our study is among the first to test the biogeographic patterns at genus, a more meticulous taxonomic level, involving 1668 islands, a scale not previously attempted. Besides, our study is among the first attempt to explore nestedness at both whole and portion level. Our study on herpetofaunas thus fills in a significant gap, contributes to the recent heated disputes on the SIE theory (Dengler 2010; Tjørve and Tjørve 2011; Triantis and Sfenthourakis 2012; Wang et al. 2012) and nestedness (Matthews et al. 2015), and expands our horizons on nestedness at different spatial scales.

We found piecewise regressions with three segments were better than those with two segments, suggesting there are three different SAR patterns that resulted from two area thresholds. Our findings strongly support the theory proposed by Lomolino and Weiser (2001) and Rosenzweig (2004), who have argued that there are three biological scales of species–area curve with three corresponding dominant processes of species addition. Our results are convincible as our analyses meet the criteria generally considered necessary for the unambiguous detection of SIE (Dengler 2010): (1) including not only the intermediate and large islands but also hundreds of small islands that have few or no herpetofaunal records; (2) comparing most relevant models; (3) selecting models in the same *S*‐space (log *S*‐space); and (4) accounting for model complexity using AIC when comparing models with different numbers of parameters. The two‐slope approach is always better than the left‐horizontal with one threshold approach; the three‐slope approach is also likely to be better than the left‐horizontal with two thresholds approach (except for *Peltophryne* frogs) at explaining the data. This may be due to the fact that the discontinuous functions are more flexible than the two left‐horizontal functions, and the flexibility may in turn improve their fitness to compensate the increment of parameter number. Although the left‐horizontal with two thresholds function is selected as the best model for *Peltophryne* frogs, the slope at islands of intermediate size (*z*_1_) is unrealistically higher than the slope at large islands (*z*_2_). And this may be due to small number of islands being occupied (*n *=* *12), an inadequate sample size for robust modeling (Chase and Bown 1997).

Currently, piecewise regressions with two segments have been widely used in SIE studies (Lomolino and Weiser 2001; Gentile and Argano 2005; Dengler 2010; Wang et al. 2012; Matthews et al. 2014; Morrison 2014). However, three SAR patterns with three different *z* values cannot be fully depicted by two segments, and the two‐segment regression is likely to cause islands of intermediate size to split into two factions: one faction along with small islands to delimit the SIE, and the other faction along with large islands to delimit evolutionary processes. Therefore, the two‐segment regression could possibly lead to poor estimations of both slope (for the two‐slope approach) and threshold value (for both the two‐slope approach and the left‐horizontal with one threshold approach) of the SIE. And that may explain why *z*_1_ values in the three‐slope approach were much lower than those in the two‐slope approach in our study.

Although the *z*_1_ parameters (in the discontinuous functions) were significantly different from zero, the *z*_1_ values in the three‐slope function were in the range 0.003–0.080, with an average of 0.019, which is very close to zero. Three main hypotheses have been proposed to explain the SIE: First, within the range of the SIE, species richness is area independent, presumably because populations are unstable and entire fauna could be wiped out by storms, tidal surges, or other stochastic events (MacArthur and Wilson 1967; Losos 1996). Second, some habitat types are removed with the reduction of area (Niering 1963; Triantis et al. 2006), obliterating species, especially habitat specialists (Sfenthourakis and Triantis 2009). Furthermore, small islands receive greater amounts of nutrient influxes per unit area from the surrounding system than large islands, such that island area alone is not a sufficient predictor of species richness (Anderson and Wait 2001; Barret et al. 2003). However, even if small islands receive greater amounts of nutrient subsidies, there is no evidence that they can be used by herpetofaunal species; therefore, nutrient subsidies may play little role in the system. Moreover, the long human presence and high frequency of human influence continuously supply new colonists to some small islands but also transform previous land‐use types into anthropogenic biotopes such as cultivation and settlements (Sfenthourakis 1996), hosting some herpetofaunal species that coexist with humans (Raxworthy and Nussbaum 2000; Henderson and Powell 2001), counteracting the occurrence of stochastic events and the loss of critical habitat due to area reduction. And that may explain why slopes at the lower end were approaching to zero but not equal to zero.

As compared with inferior taxonomic groups, species in superior taxonomic groups greatly enhances viability in a water‐deficient and space‐and‐resource‐limited environment, lowering the extinction rates when they colonize an island and making themselves effective dispersers. This inference is consistent with the fact that superior taxonomic groups have a wider distribution, for example, 738, 571, and 119 islands for Reptiles, *Anolis* lizards, and *Eleutherodactylus* frogs vs. 124, 373, and 12 islands for Amphibians, *Sphaerodactylus* lizards, and *Peltophryne* frogs. It is clear that there are three different species–area patterns in our system. Within the first threshold (*T*_1_), species richness slightly increases with island size, likely because habitat type and habitat quality decrease as area gets smaller and smaller (Niering 1963; Losos 1996). When area is so small that species of inferior taxonomic groups cannot survive, superior taxonomic groups may still sustain viable populations, so their species richness is still area dependent. That is why the first threshold values (*T*_1_) of superior taxonomic groups are lower than that of inferior taxonomic groups. On the other hand, beyond the second threshold (*T*_2_), species richness steeply increases with island size, likely because larger islands have lower extinction rates, higher immigration rates (MacArthur and Wilson 1967), larger population size (Gilpin and Diamond 1976), higher diversity of habitats, higher coverage of each habitat type (Lomolino 1990; Lomolino and Weiser 2001), and higher chance of internal geographic isolation (Losos 1996). As compared with the inferior groups, taxa in the superior groups may have a wider distribution on an island because of the better adaptation to their environment, and thus, it is more likely to occur that unfavorable habitats among populations keep them from mating with one another or mating throughout a population is not random if the population extends over a broad geographic range. So, when area is not too large that species of inferior taxonomic groups are still governed by habitat diversity, carrying capacity, and extinction/immigration dynamics, superior taxonomic groups may have already entered into the evolutionary stage. That is why the second threshold values (*T*_2_) of superior taxonomic groups are also lower than that of inferior taxonomic groups. Our results provide evidence for the prediction made by Lomolino and Weiser (2001) who stated that the upper limit of the threshold values tended to be higher for species groups with relatively high resource requirements and low dispersal abilities.

MacArthur and Wilson (1967) stated that the range of insular *z* values was 0.20–0.35, Rosenzweig (1995) later narrowed it to 0.25–0.33. However, beyond the second threshold (*T*_2_), the *z* values (0.42–0.94) were very high according to the three‐slope function, reflecting species diversity is governed by not only the dynamics of immigrations but also considerable and rapid in situ speciation (Lomolino 2000). This result is consistent with that of Losos and Schluter (2000), who suggested that within‐island speciation exceeds immigration as a source of new species on large islands, whereas speciation is rare on small islands. This result is also consistent with the findings of our previous studies on the same set of 1668 islands, which indicate the total *β*‐diversity can be explained largely by in situ speciation rather than island size (Gao and Perry, in submission). Speciation occurring within large islands will in turn lead to species endemism in large islands, decreasing the likelihood of nestedness and increasing the likelihood of antinestedness in a system (Whittaker and Fernández‐Palacios 2007). Apart from within‐island speciation, human introductions have become a new mode of entering the region for some species, many of which are not native to the West Indies. For instance, *Anolis carolinensis* (native to USA) has arrived on Anguilla with the development of tourism (Eaton et al. 2001). The human‐mediated species introduction is much likely to be island specific and may decrease the nestedness as well.

Although the whole system is unlikely to be nested, some portions within the system may still be nested as they get a relatively high NODF score, and such a high NODF score may correlate with a high matrix fill (Almeida‐Neto et al. 2008). However, this relationship has been argued not an analytical artifact but simply a consequence of the concept of nestedness, because matrix fill corresponds to the degree of species occupancy (Almeida‐Neto et al. 2008). There are many factors representing different mechanisms at explaining nestedness. According to the classical island biogeography theory (MacArthur and Wilson 1967), the probability of immigration increases as island isolation decreases, and the probability of extinction increases as island area decreases. A high degree of nestedness in a matrix in which fragments are sorted by area suggests the importance of extinction. In this case, species with larger area requirements have a greater risk of extinction, and thus, a predictable sequence of extinction occurs in relation to island size (Patterson and Atmar 1986). A high degree of nestedness in a matrix sorted by isolation indicates the importance of immigration. In this case, nestedness is due to predictable dispersal limitation, such that nestedness occurs due to differential immigration to islands (Kadmon 1995). In addition to area and isolation, some other factors may also be important in producing nested patterns, such as habitat nestedness (Honnay et al. 1999), habitat quality (Hylander et al. 2005; Triantis and Bhagwat 2011), and disturbance (Fleishman and Murphy 1999; Wang et al. 2013). In this respect, our finding shines light on the further research to determine and compare the factors producing nested patterns at different areas.

## Conclusions

Although piecewise regressions with two segments have been widely used in SIE detection studies, they cannot clearly delimit three SAR patterns and may cause poor estimations for both slope and threshold value of the SIE. Our findings suggest previous SIE detection studies conducted by the two‐segment piecewise regression method should be reanalyzed.

No matter the doubts about the existence of the SIE, the threshold value, where the slope changes, may be important for a successful application of island theory to conservation biogeography. Apart from area, it offers opportunity to assess variables such as habitat diversity, productivity, island age, energy, and environmental heterogeneity (Whitehead and Jones 1969; Anderson and Wait 2001; Tjørve and Tjørve 2011; Triantis and Bhagwat 2011) that may predict species richness within the limits of the first threshold value. On the other hand, speciation may become the dominant process adding to the species richness of assemblages beyond the limits of the second threshold value (Losos and Schluter 2000), so the identification of such size threshold shines light on conservation biogeography over evolutionary timescales. Moreover, the comparison of threshold values will help evaluate resource requirement, dispersal ability, as well as environmental adaptation among taxa. And this in turn will help set up taxon‐specific conservation planning.

A strong degree of nestedness implies that most species could be represented by conserving the largest (habitat or true) island. However, the low degree of nestedness shown in our result is consistent with the findings of our previous studies on the same set of 1668 islands, which indicate that species richness of the largest island fail to reach half the number of species pool (Gao and Perry, in submission). Contrary to the concept of protecting the largest reserve, we conclude that an array of reserves of different size and endemism could contribute to the maximal diversity in a region.

## Conflict of Interest

None declared.

## Supporting information


**Figure S1.** The iterative process used in left‐horizontal with one threshold approach to determine the break point for each taxonomic group.
**Figure S2.** The iterative process used in two‐slope approach to determine the break point for each taxonomic group.
**Figure S3.** The iterative process used in left‐horizontal with two thresholds approach to determine the break points for each taxonomic group.
**Figure S4.** The iterative process used in three‐slope approach to determine the break points for each taxonomic group.
**Figure S5.** Detailed display of each model function fitted to each taxonomic group.

**Table S1.** Presence–absence matrix of *Anolis* lizards.

**Table S2.** Presence–absence matrix of *Sphaerodactylus* lizards.

**Table S3.** Presence–absence matrix of *Eleutherodactylus* frogs.

**Table S4.** Presence–absence matrix of *Peltophryne* frogs.
