# Supplementary material for: Detecting the small island effect and nestedness of herpetofauna of the West Indies
Source: Ecol Evol. 2016 Jul 5;6(15):5390–403. doi: 10.1002/ece3.2289 (PMC4984512; doi:10.1002/ece3.2289)
Supplement: Supplementary file 1 — Figure S1. The iterative process used in left‐horizontal with one threshold approach to determine the break point for each taxonomic group. Figure S2. The iterative process used in two‐slope approach to determine the break point for each taxonomic group. Figure S3. The iterative process used in left‐horizontal with two thresholds approach to determine the break points for each taxonomic group. Figure S4. The iterative process used in three‐slope approach to determine the break points for each taxonomic group. Figure S5. Detailed display of each model function fitted to each taxonomic group. [file ECE3-6-5390-s001.docx]

**Figure S1.** The iterative process used in left-horizontal with one threshold approach to determine the breakpoint for each taxonomic group. The breakpoint that returns a minimal residual sum of squares (*RSS*) was chosen.

| 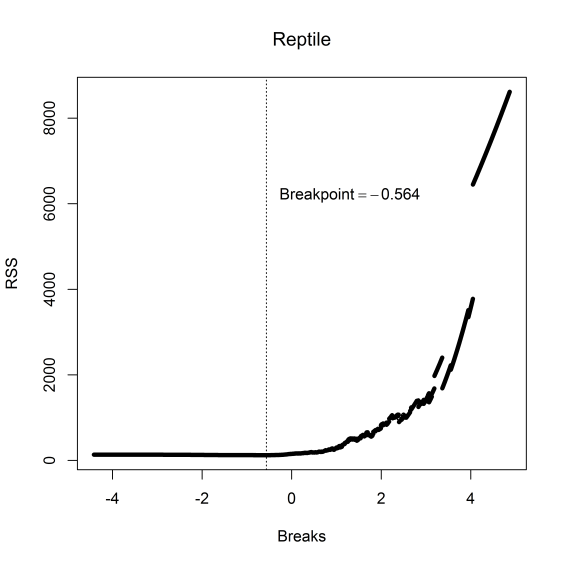 | 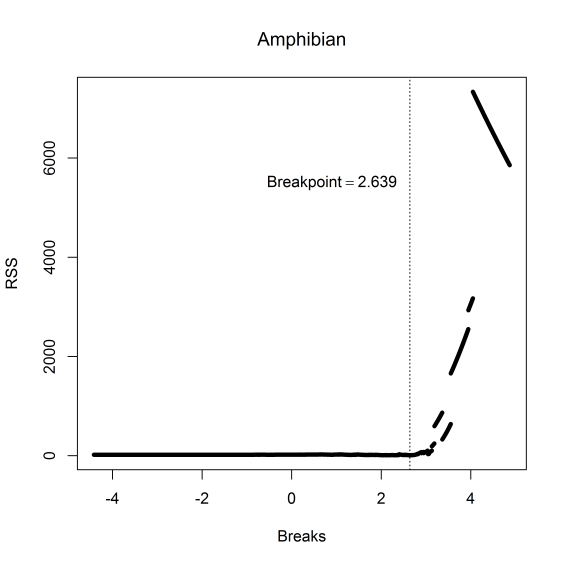 |
| --- | --- |
| 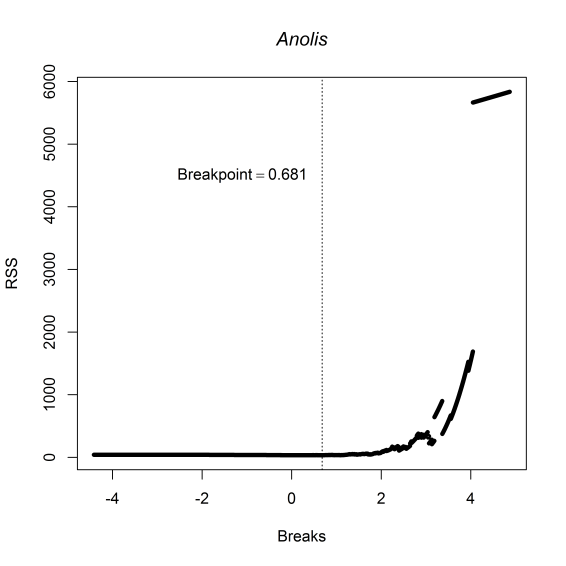 | 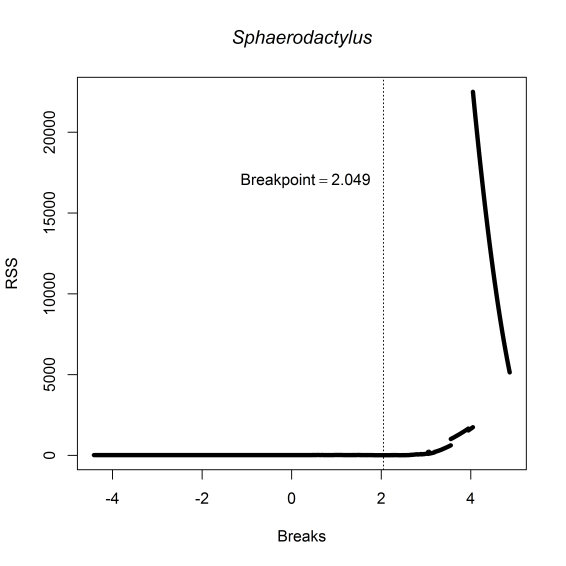 |
| 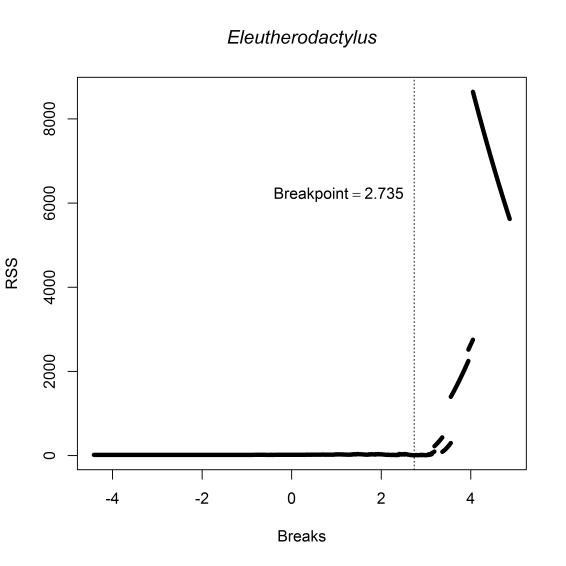 | 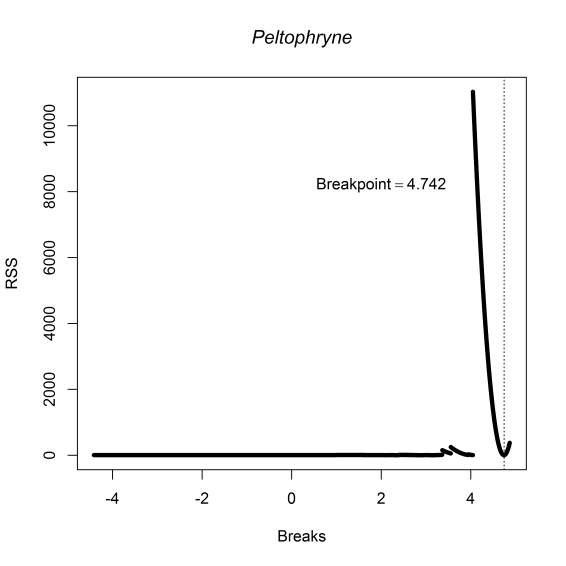 |

**Figure S2.** The iterative process used in two-slope approach to determine the breakpoint for each taxonomic group. The breakpoint that returns a minimal residual sum of squares (*RSS*) was chosen.

| 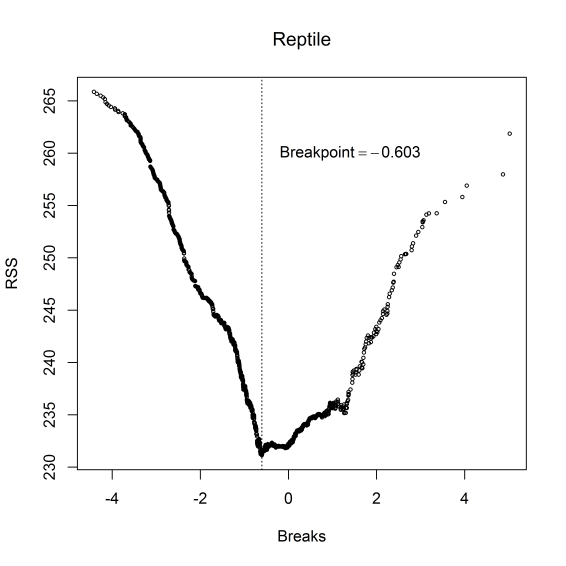 | 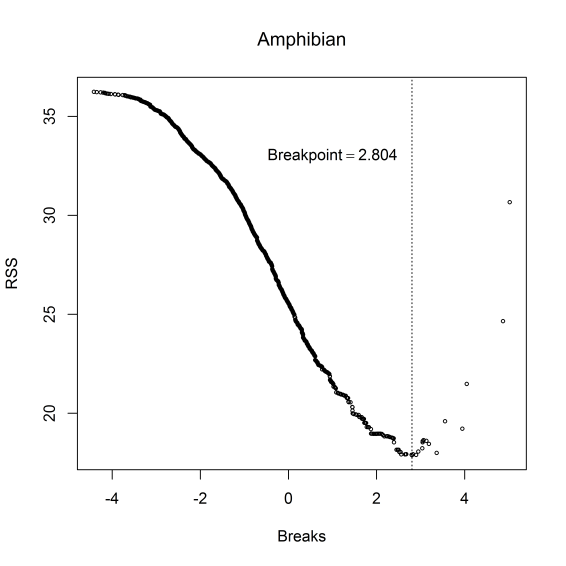 |
| --- | --- |
| 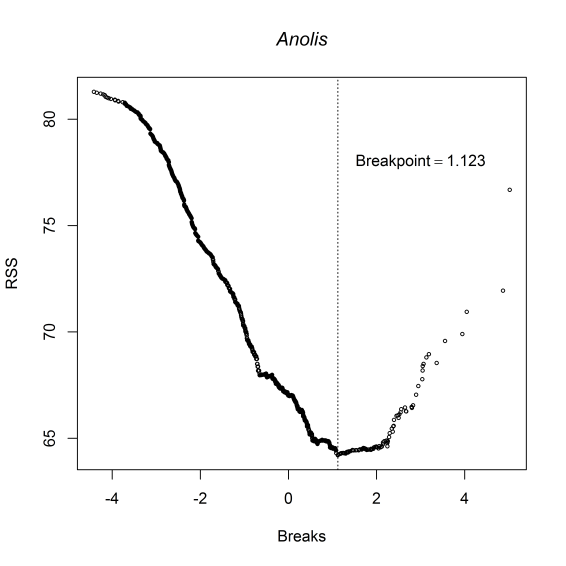 | 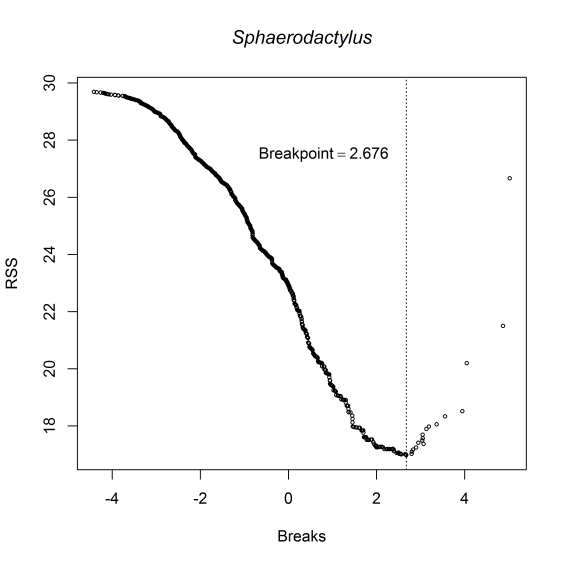 |
| 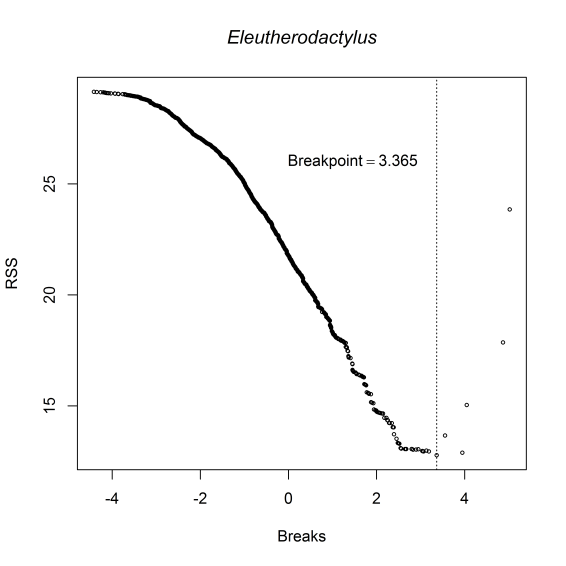 | 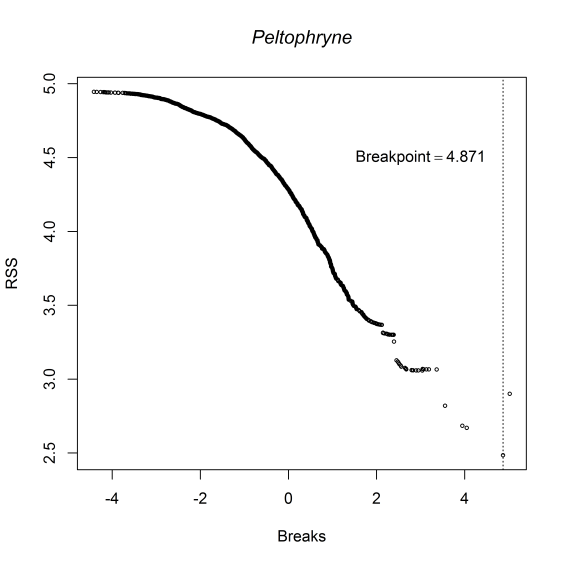 |

**Figure S3.** The iterative process used in left-horizontal with two thresholds approach to determine the breakpoints for each taxonomic group. The breakpoints that return a minimal residual sum of squares (*RSS*) were chosen. The second breakpoint was obtained prior to the first one.

| 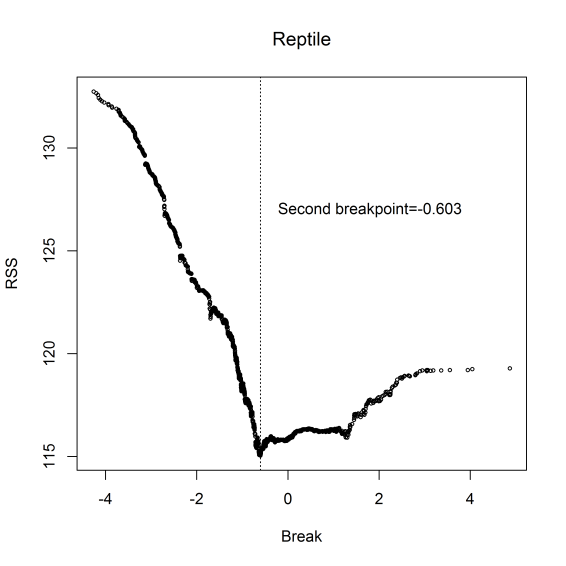 | 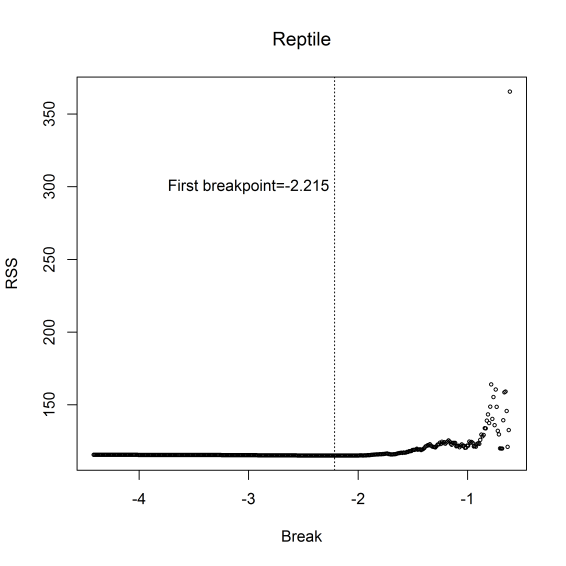 |
| --- | --- |
| 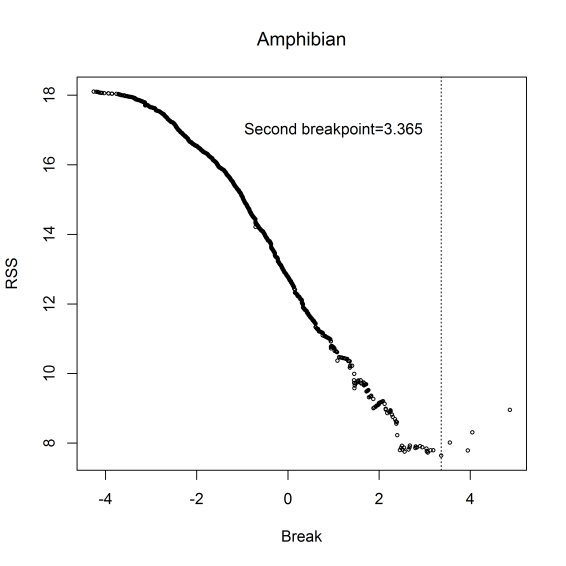 | 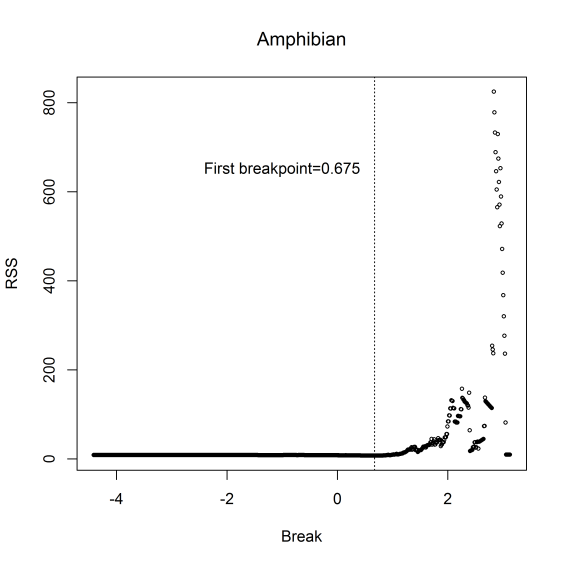 |
| 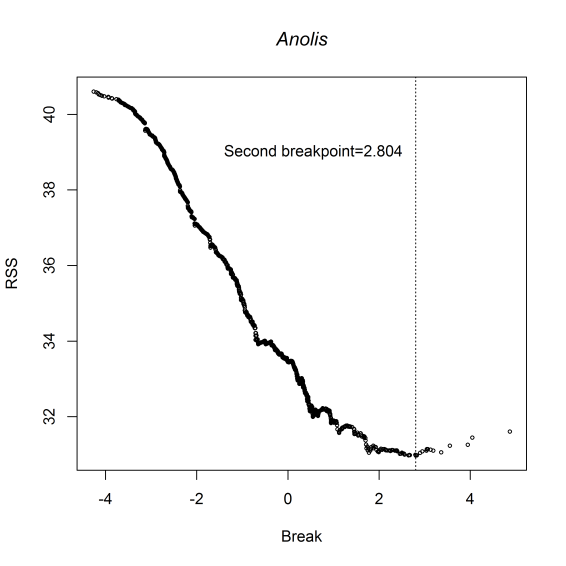 | 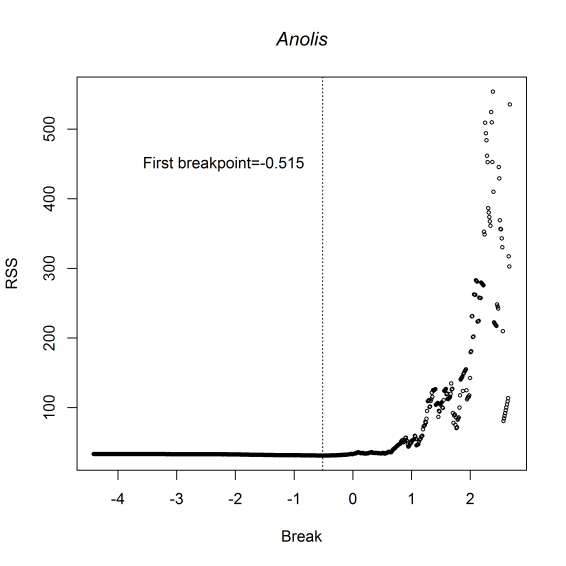 |
| 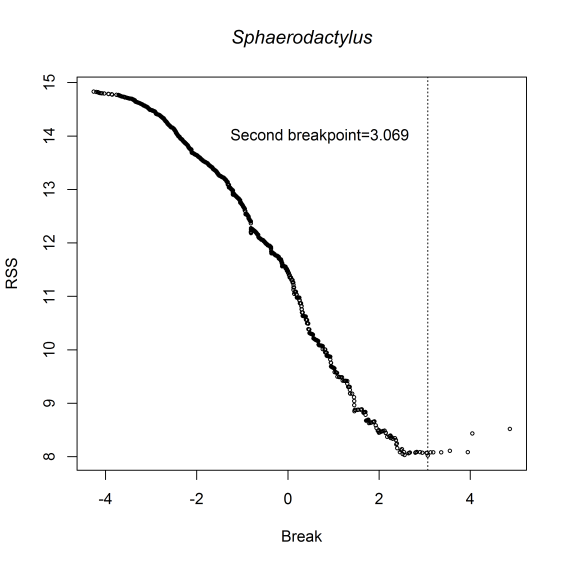 | 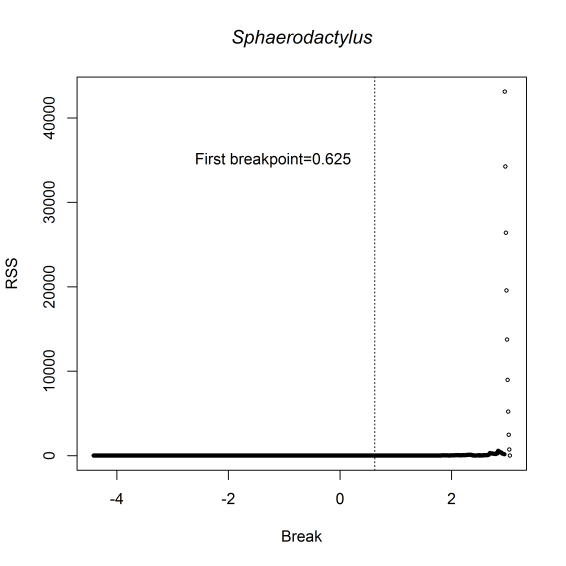 |
| 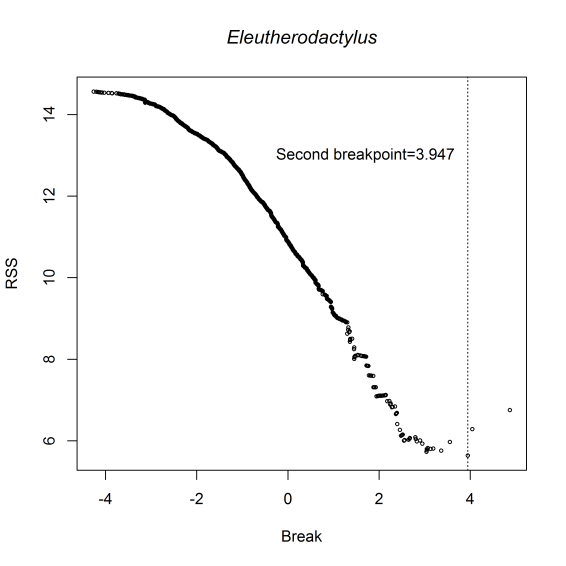 | 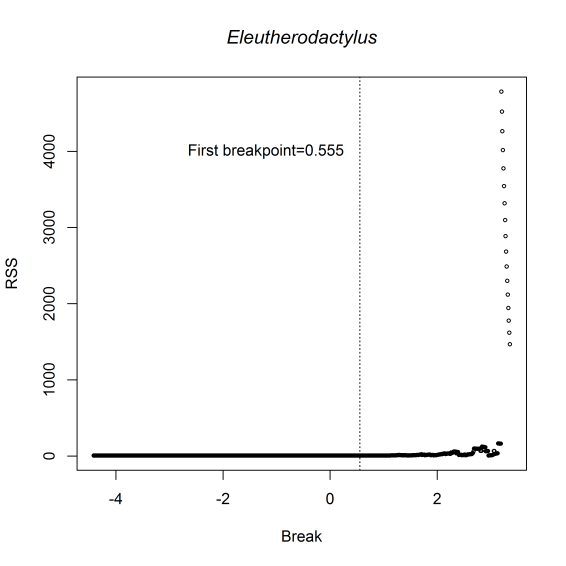 |
| 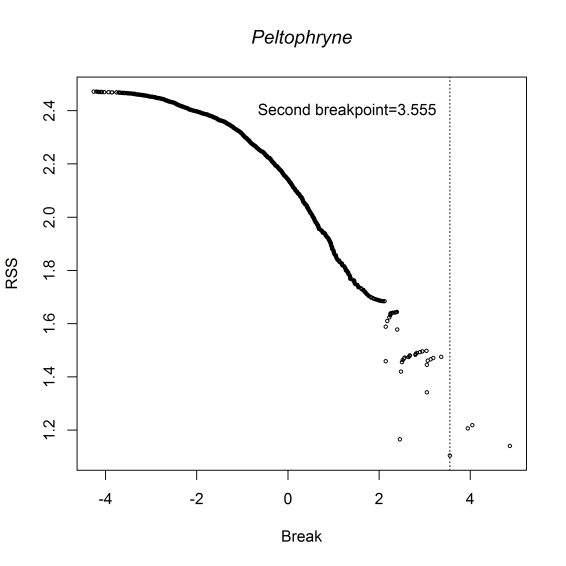 | 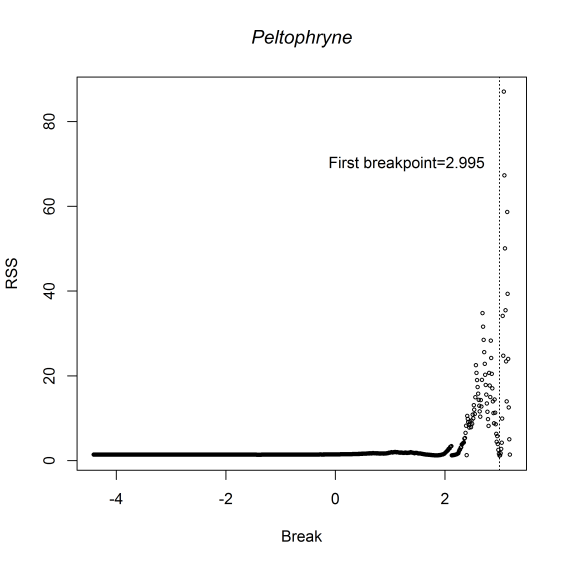 |

**Figure S4.** The iterative process used in three-slope approach to determine the breakpoints for each taxonomic group. The breakpoints that return a minimal residual sum of squares (*RSS*) were chosen. The first breakpoint was obtained prior to the second one.

| 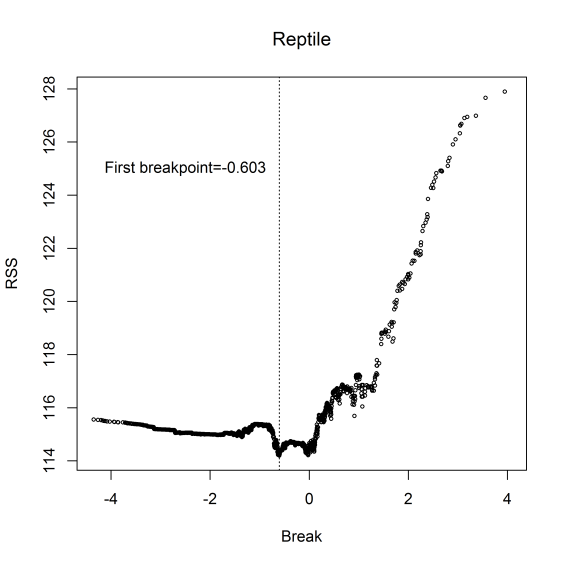 | 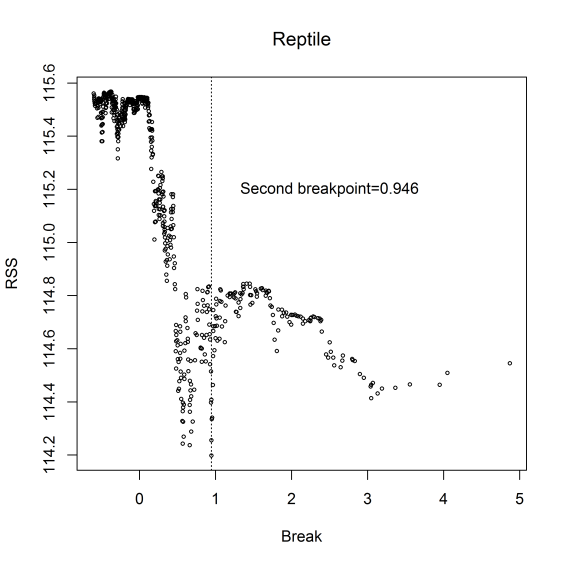 |
| --- | --- |
| 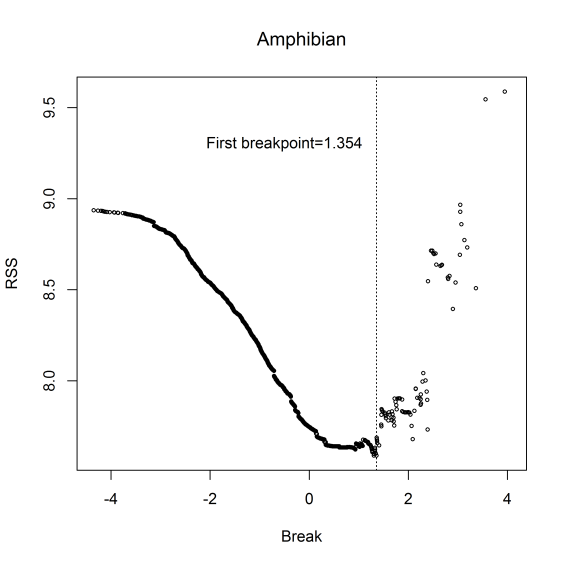 | 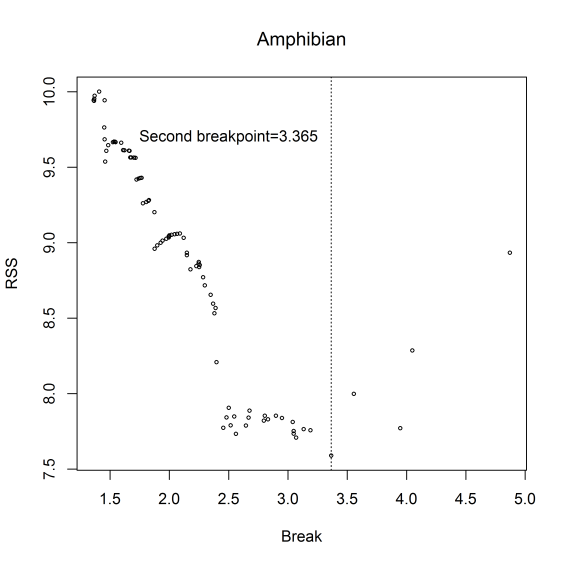 |
| 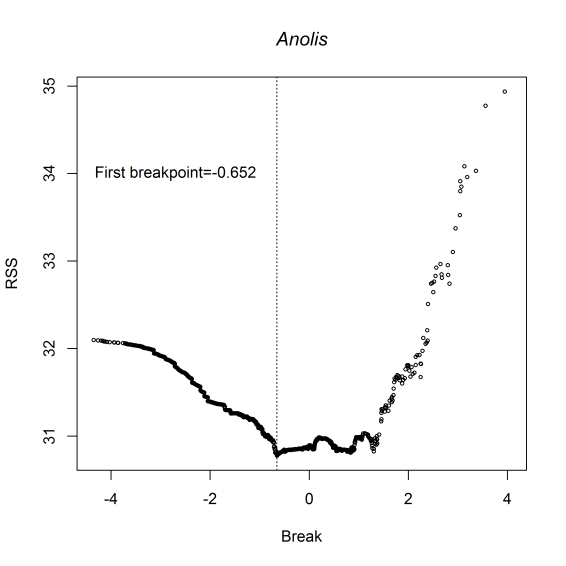 | 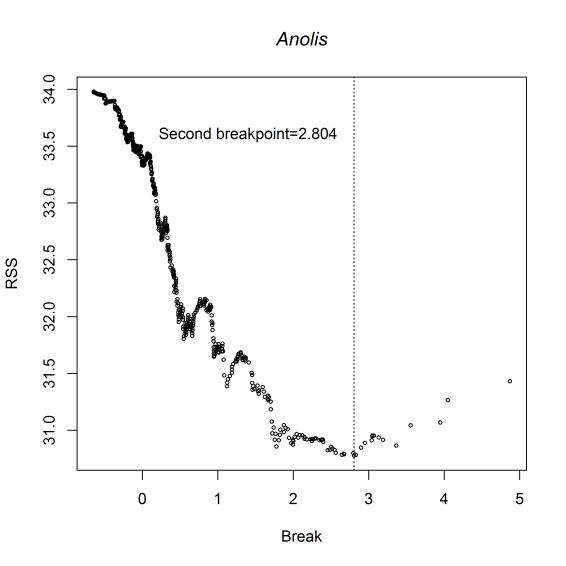 |
| 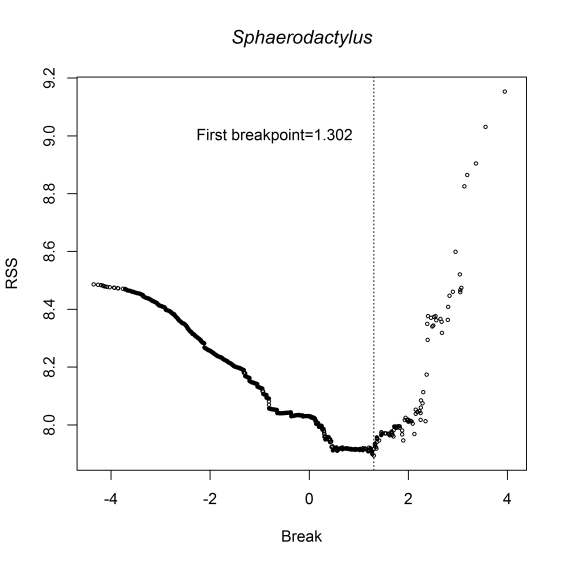 | 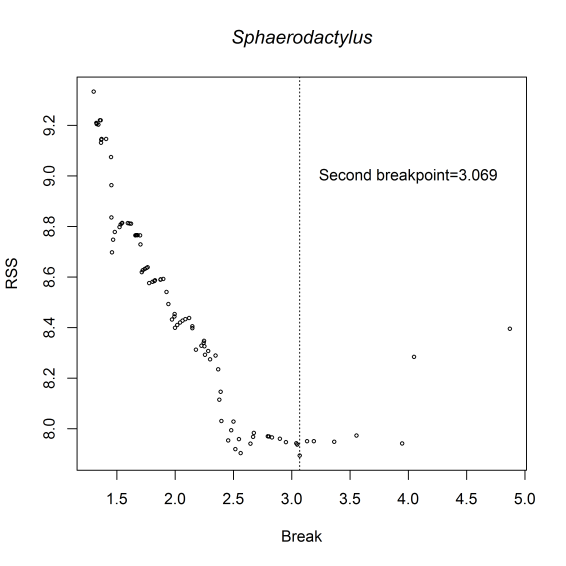 |
| 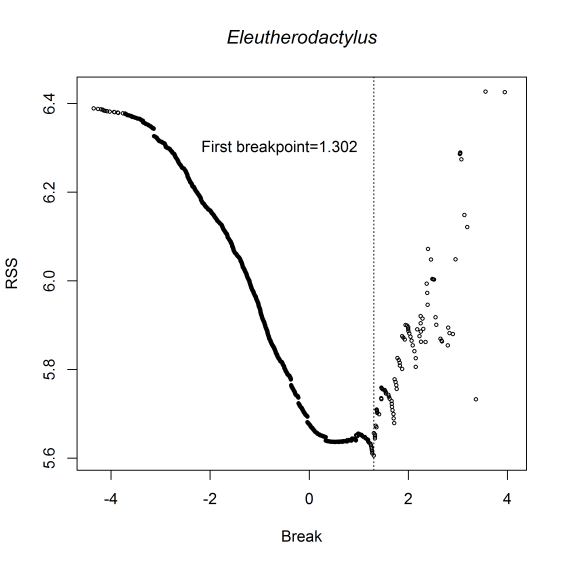 | 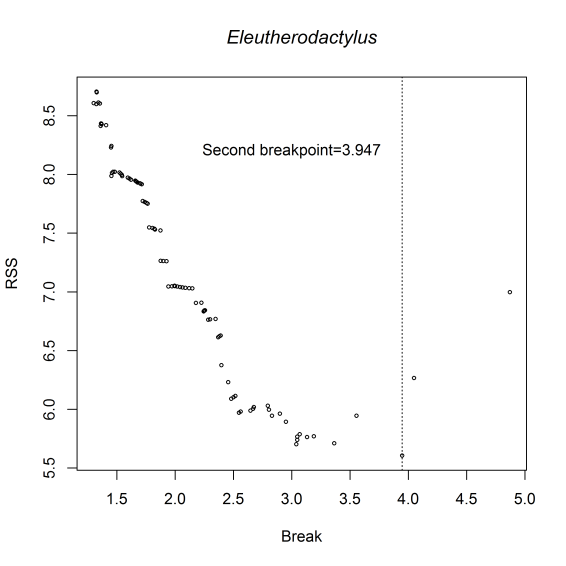 |
| 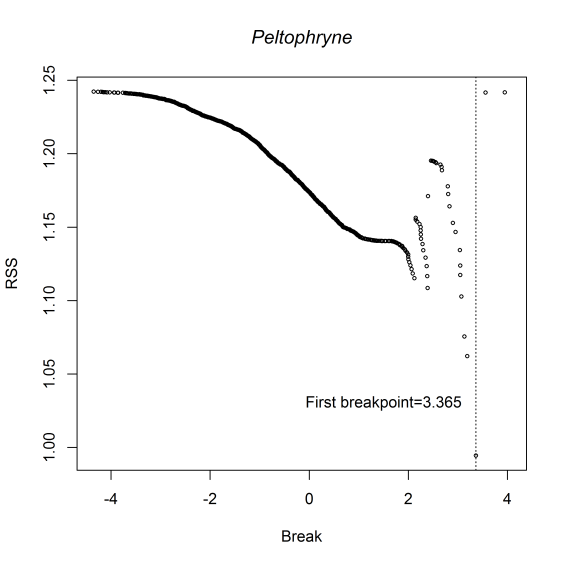 | 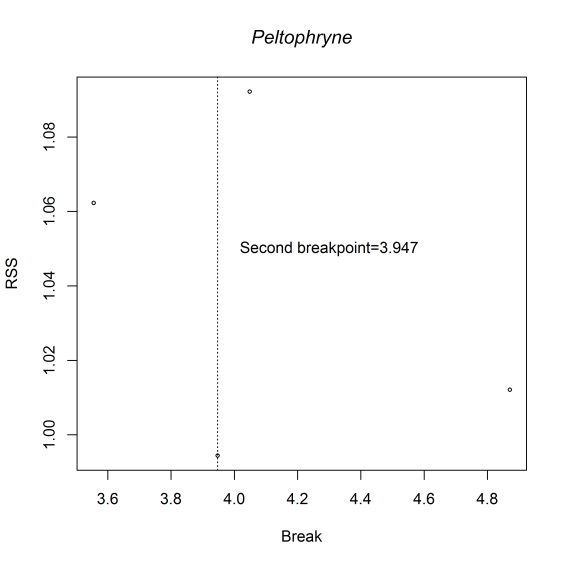 |

**Figure S5.** Detailed display of each model function fitted to each taxonomic group. Black, blue, red, green, and orange lines indicate linear, two-slope, left-horizontal with one threshold, three-slope, and left-horizontal with two thresholds, respectively.

| 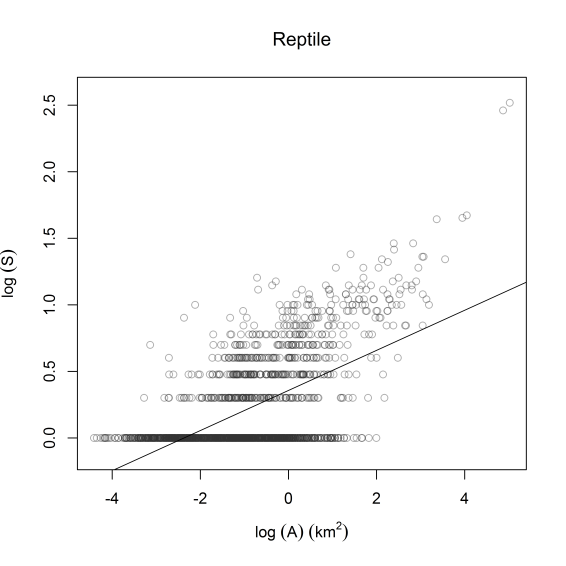 |  |
| --- | --- |
| 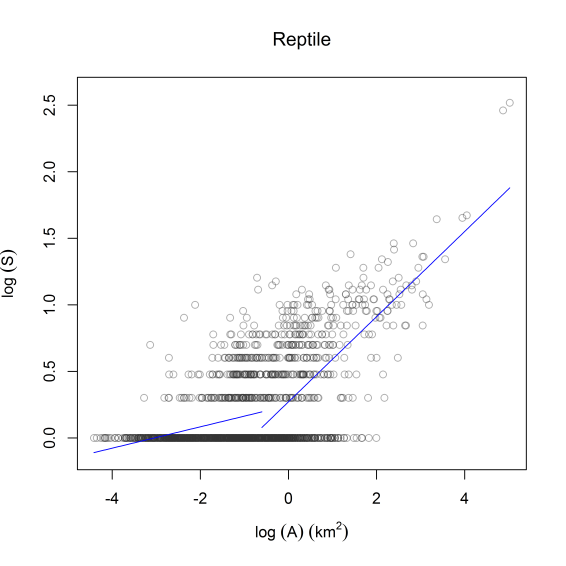 | 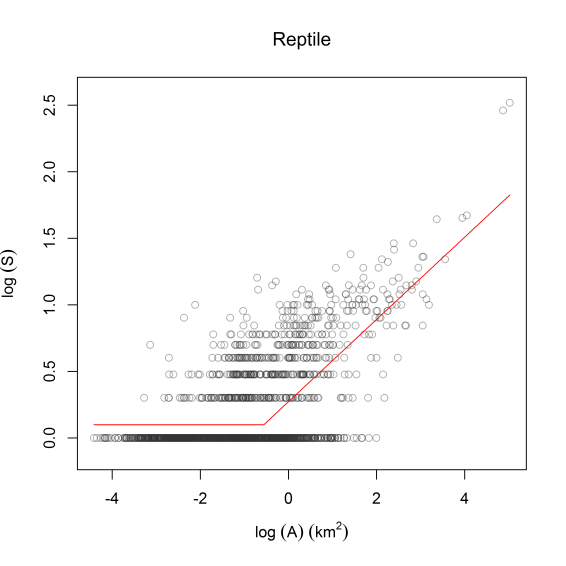 |
| 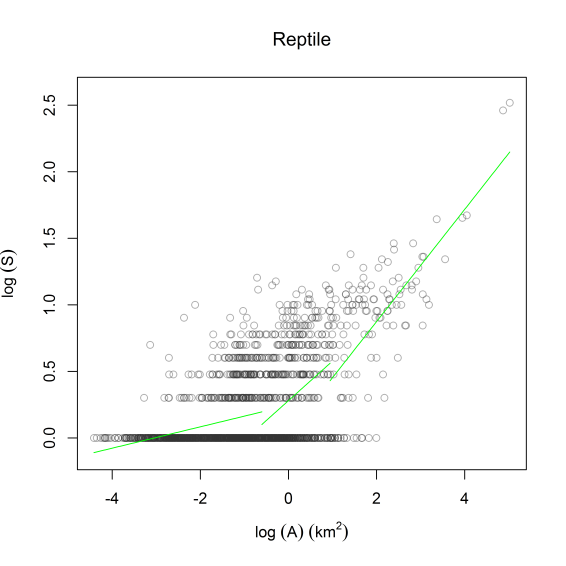 | 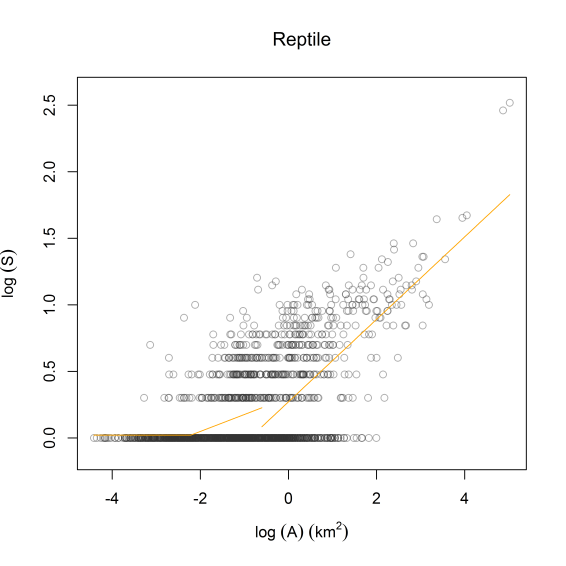 |

| 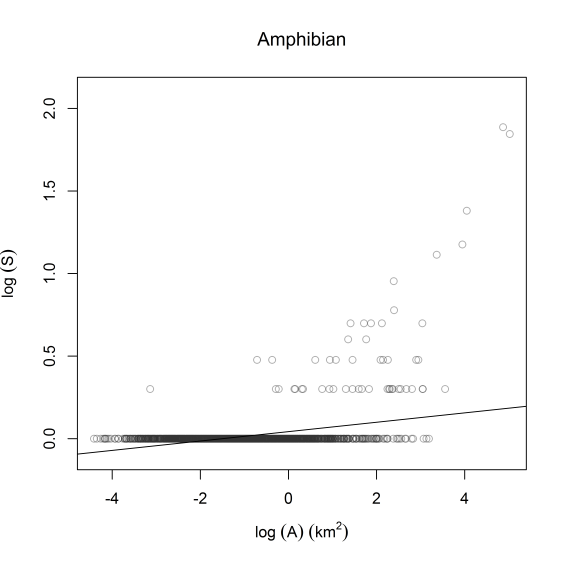 |  |
| --- | --- |
| 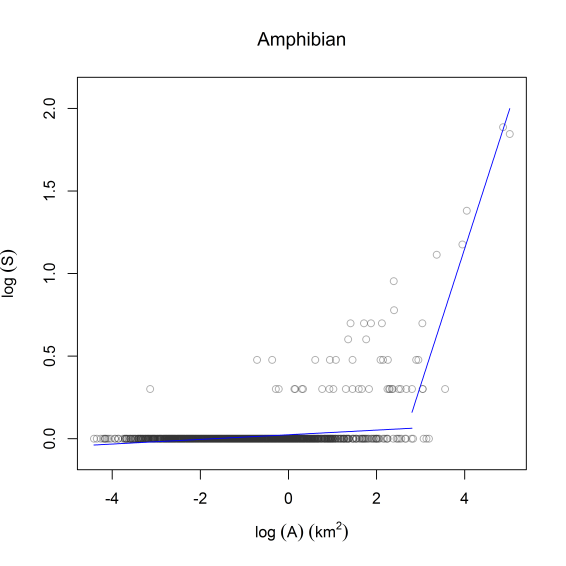 | 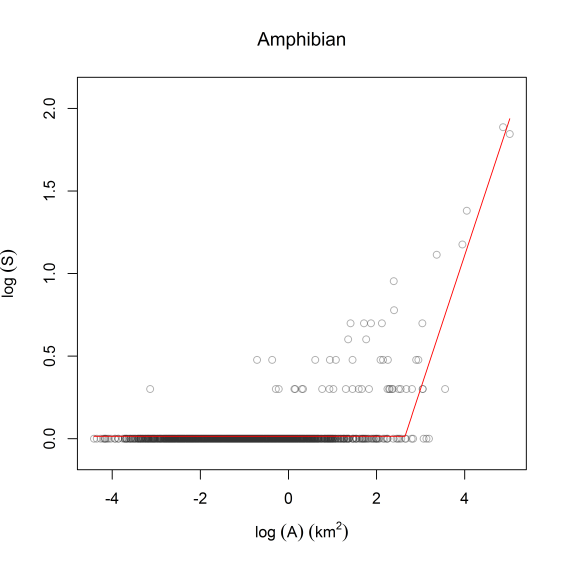 |
| 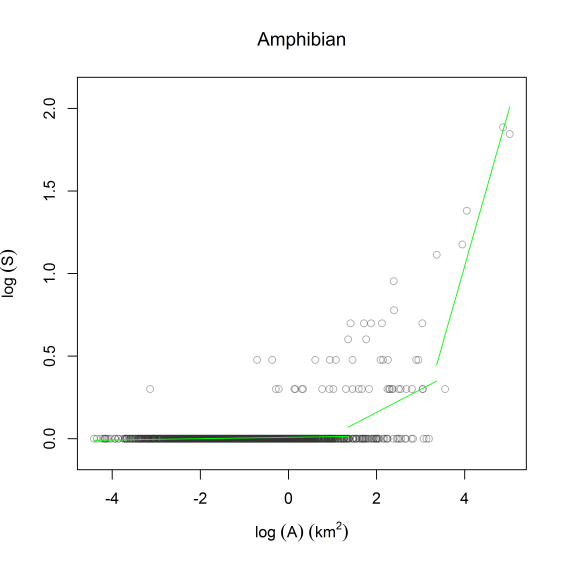 | 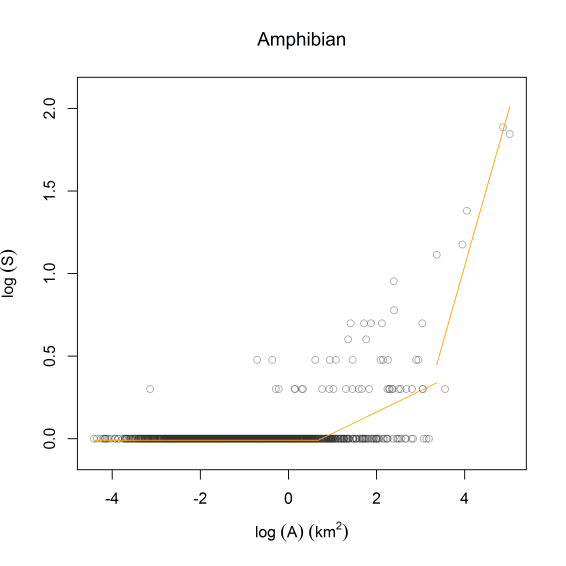 |

| 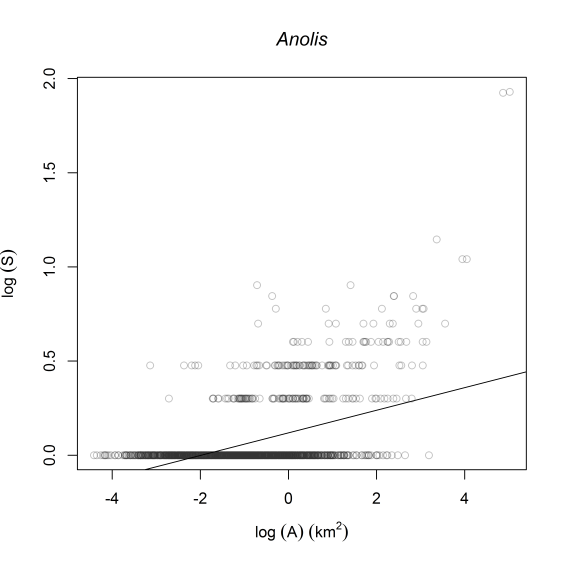 |  |
| --- | --- |
| 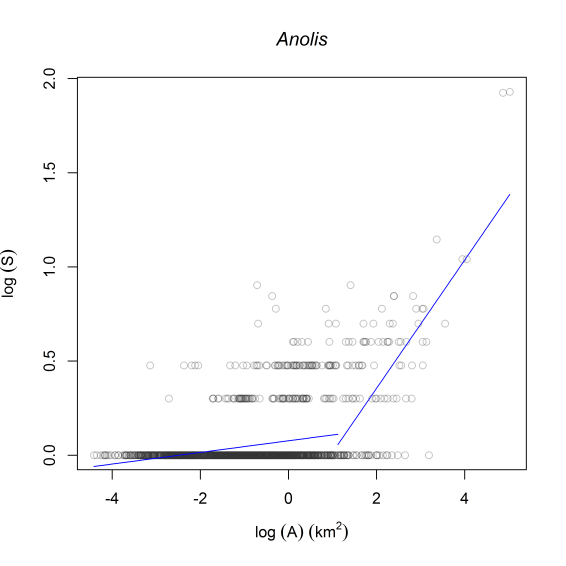 | 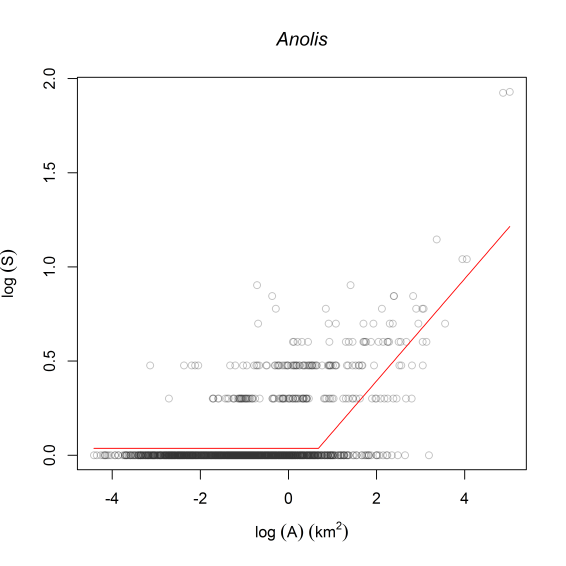 |
| 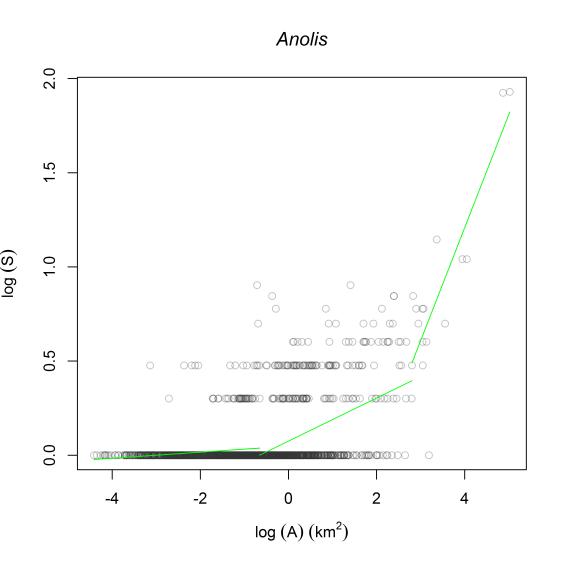 | 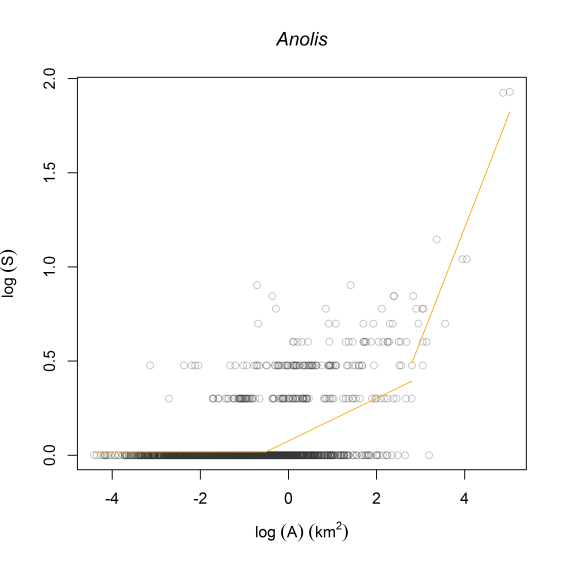 |

| 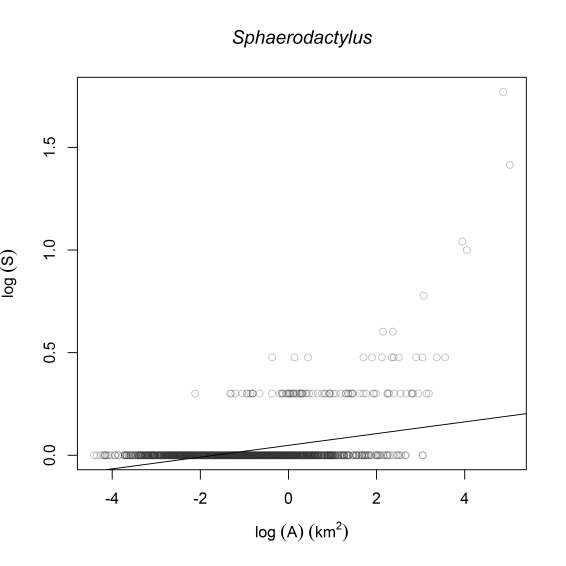 |  |
| --- | --- |
| 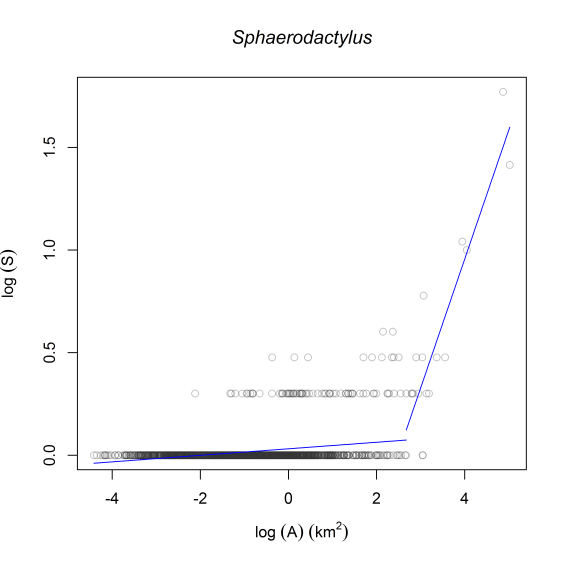 | 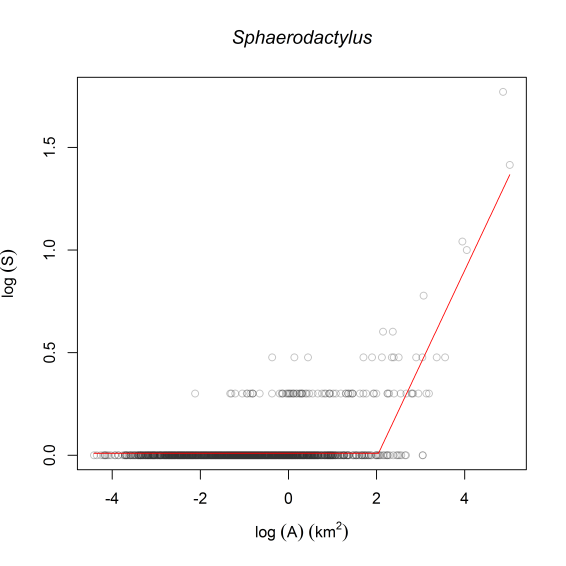 |
| 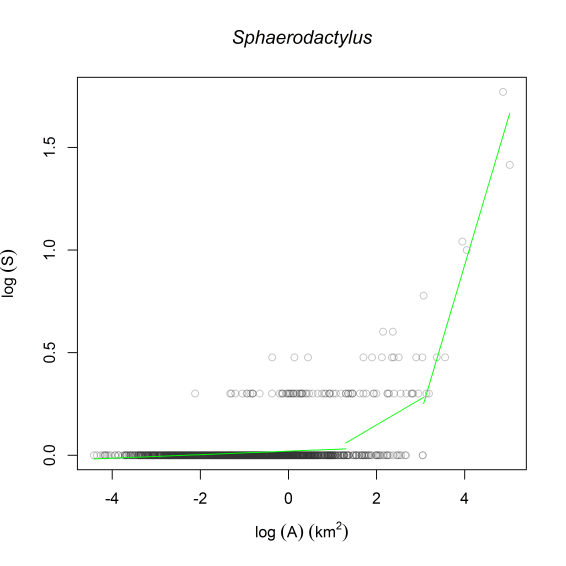 | 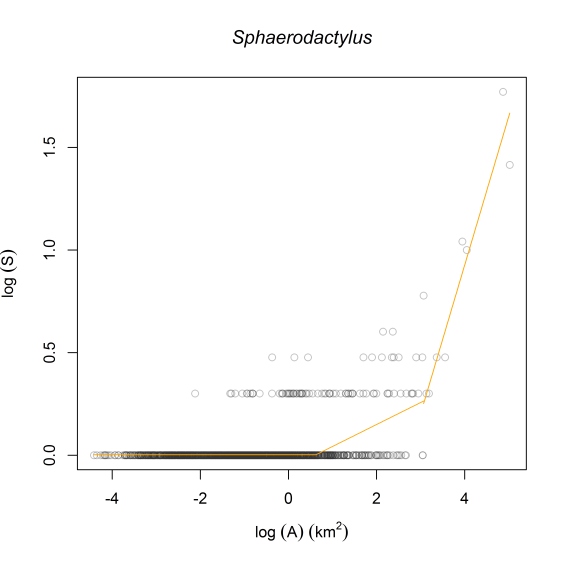 |

| 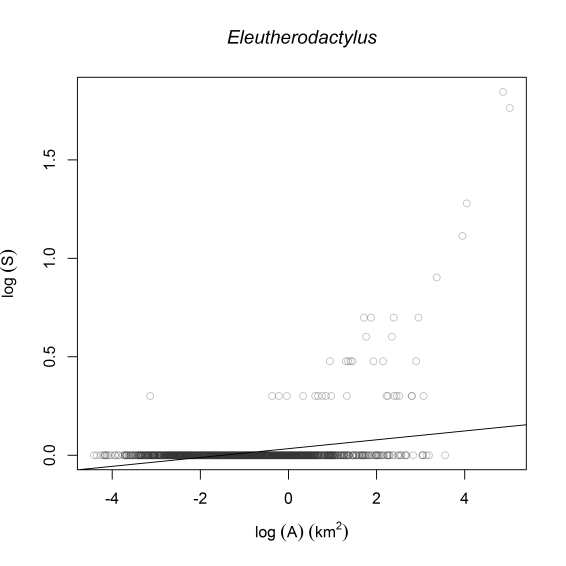 |  |
| --- | --- |
| 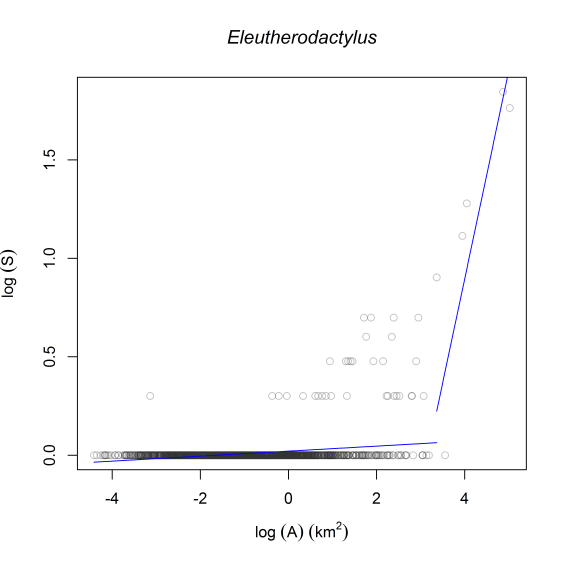 | 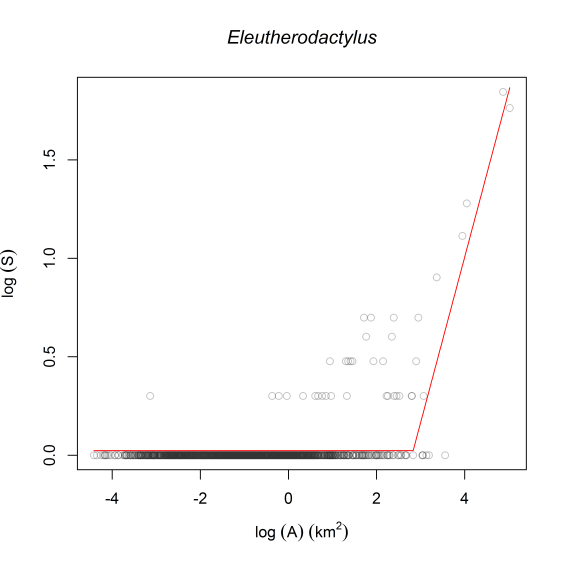 |
| 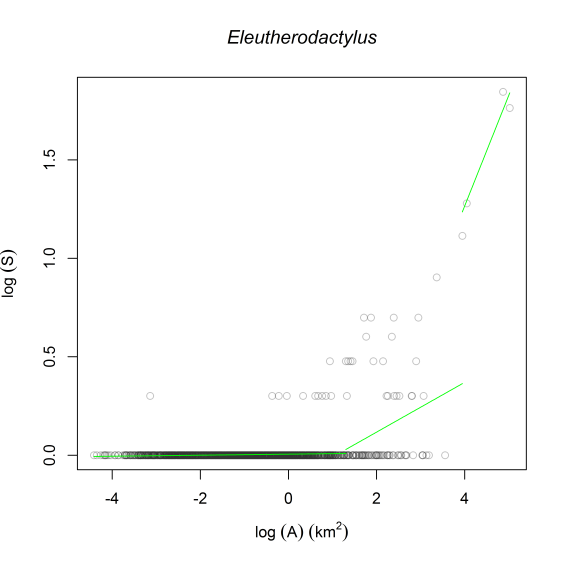 | 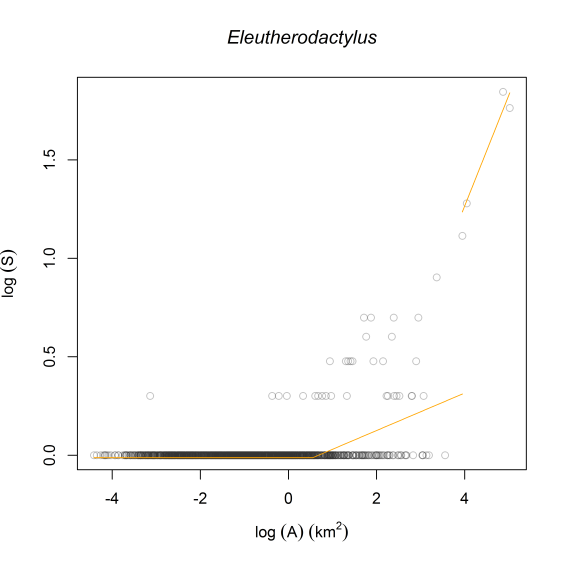 |

| 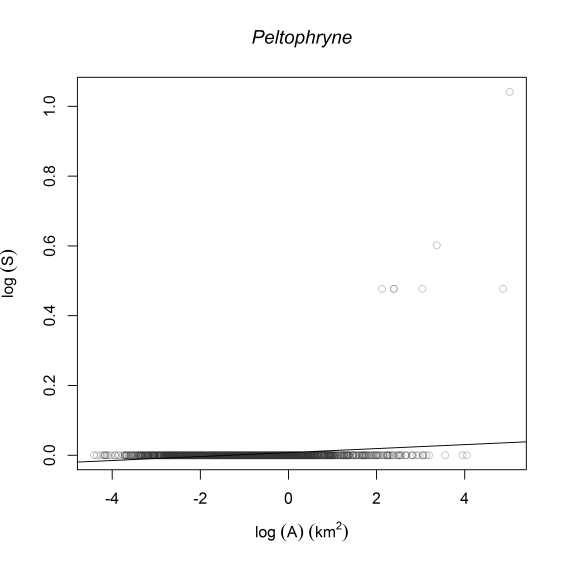 |  |
| --- | --- |
| 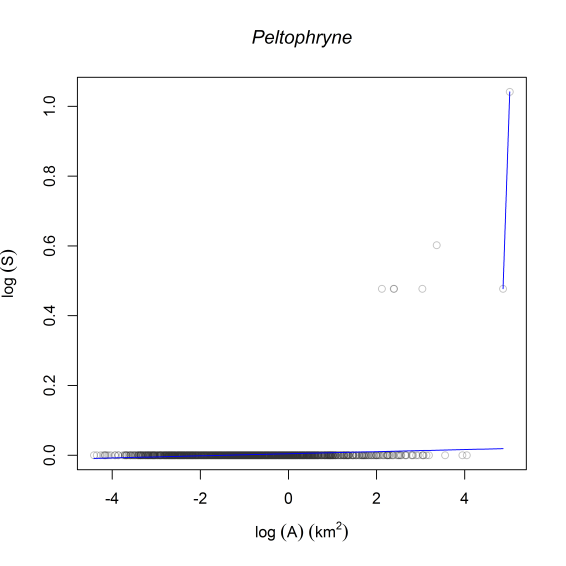 | 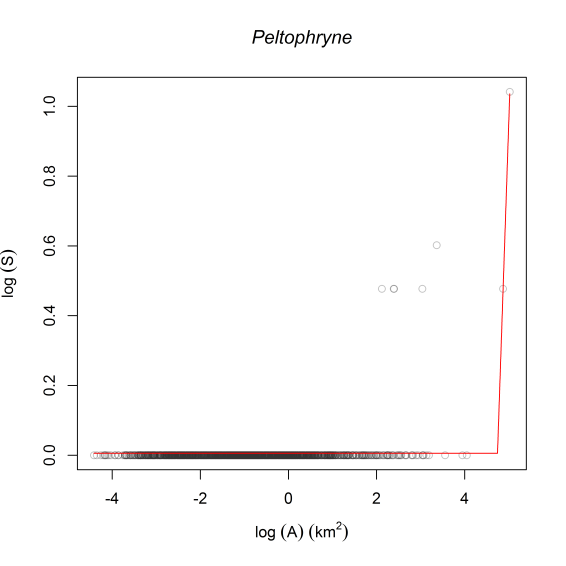 |
| 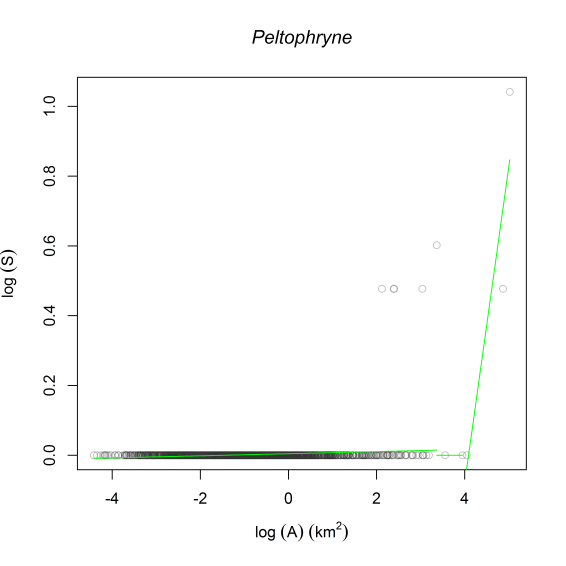 | 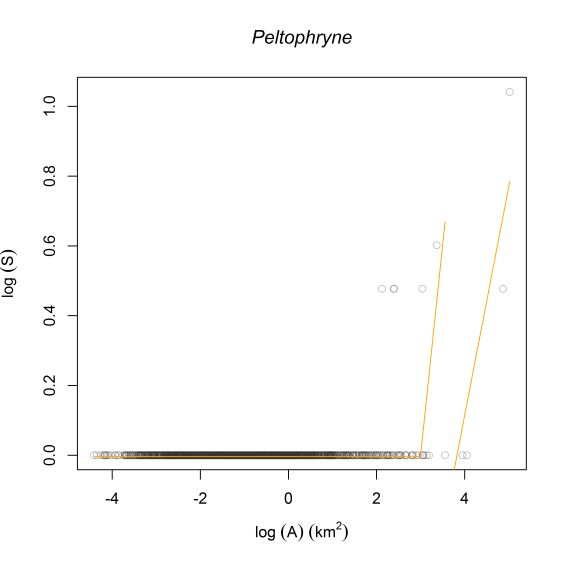 |
